# A cellular reporter system to evaluate endogenous fetal hemoglobin induction and screen for therapeutic compounds

**DOI:** 10.1002/hem3.139

**Published:** 2024-08-06

**Authors:** Thijs C. J. Verheul, Nynke Gillemans, Kerstin Putzker, Rezin Majied, Tingyue Li, Memnia Vasiliou, Bert Eussen, Annelies de Klein, Wilfred F. J. van IJcken, Emile van den Akker, Marieke von Lindern, Joe Lewis, Ulrike Uhrig, Yukio Nakamura, Thamar van Dijk, Sjaak Philipsen

**Affiliations:** ^1^ Department of Cell Biology Erasmus MC Rotterdam The Netherlands; ^2^ EMBL Chemical Biology Core facility Heidelberg Germany; ^3^ Department of Clinical Genetics Erasmus MC Rotterdam The Netherlands; ^4^ Genomics Core Facility, Erasmus MC Rotterdam The Netherlands; ^5^ Sanquin Research Amsterdam The Netherlands; ^6^ RIKEN BioResource Center Tsukuba Ibaraki Japan; ^7^ Present address: Anavo Therapeutics Heidelberg Germany

## Abstract

Reactivation of fetal hemoglobin expression alleviates the symptoms associated with β‐globinopathies, severe hereditary diseases with significant global health implications due to their high morbidity and mortality rates. The symptoms emerge following the postnatal transition from fetal‐to‐adult hemoglobin expression. Extensive research has focused on inducing the expression of the fetal γ‐globin subunit to reverse this switch and ameliorate these symptoms. Despite decades of research, only one compound, hydroxyurea, found its way to the clinic as an inducer of fetal hemoglobin. Unfortunately, its efficacy varies among patients, highlighting the need for more effective treatments. Erythroid cell lines have been instrumental in the pursuit of both pharmacological and genetic ways to reverse the postnatal hemoglobin switch. Here, we describe the first endogenously tagged fetal hemoglobin reporter cell line based on the adult erythroid progenitor cell line HUDEP2. Utilizing CRISPR‐Cas9‐mediated knock‐in, a bioluminescent tag was integrated at the *HBG1* gene. Subsequent extensive characterization confirmed that the resulting reporter cell line closely mirrors the HUDEP2 characteristics and that the cells report fetal hemoglobin induction with high sensitivity and specificity. This novel reporter cell line is therefore highly suitable for evaluating genetic and pharmacologic strategies to induce fetal hemoglobin. Furthermore, it provides an assay compatible with high‐throughput drug screening, exemplified by the identification of a cluster of known fetal hemoglobin inducers in a pilot study. This new tool is made available to the research community, with the aspiration that it will accelerate the search for safer and more effective strategies to reverse the hemoglobin switch.

## INTRODUCTION

Each year, nine million babies are born around the world with a congenital or genetic disease. Twenty‐five percent of these newborns suffer from just five conditions.^1^ Among these are the globinopathies, a group of monogenetic diseases including sickle cell disease (SCD) and β‐thalassemia. In these diseases, mutations affect adult β‐globin, and thus, symptoms arise after the postnatal switch from fetal (α_2_,γ_2_) to adult (α_2_,β_2_) hemoglobin expression.^2^ The importance of the hemoglobin switch (from γ to β) in marking the onset of symptoms was already observed in 1948.^3^ With this notion, the idea emerged that the re‐activation of fetal hemoglobin (HbF) expression would provide an effective treatment for these patients. In the 1960s, the discovery of Greek families with β‐thalassemia in combination with a condition called hereditary persistence of fetal hemoglobin (HPFH) proved that HbF expression ameliorates the symptoms of β‐thalassemia.^4^ Reactivation of HbF expression has ever since been a focus of drug discovery efforts for the treatment of patients with β‐globinopathies.^5^, ^6^ With the emergence of CRISPR‐based genetic therapy, reactivation of HbF expression is a promising approach that has already been tested in patients.^7^, ^8^, ^9^ In the laboratory setting, an easy‐to‐use benchtop assay that reports the activation of HbF expression would be helpful when evaluating innovations such as new nuclease enzymes or cell delivery methods.

As an alternative to genetic therapy, pharmacological induction of HbF would provide a more accessible treatment option to the majority of the patient population. Eighty percent of the ~350,000 babies born annually with severe β‐globinopathy live in sub‐Saharan Africa.^10^ Currently, hydroxyurea (HU) is the only Food and Drug Administration‐approved HbF inducer.^11^ Unfortunately, the effects of HU are variable, with some patients still developing symptoms and others displaying no significant improvement. In high‐income countries, these patients would be candidates for allogeneic bone marrow transplantation or genetic therapy (e.g., the 13‐year‐old transfusion‐dependent boy with SCD showing no response to HU described by Badat et al.^12^), while in low‐ and middle‐income countries, novel pharmacological inducers of HbF could be life saving. Over the years, many compounds have been tested, and many proteins involved in the hemoglobin switch, such as transcription factors and epigenetic enzymes, have been identified as therapeutic targets.^13^ To date, very few potent inducers of HbF have been identified, in particular when toxicity and long‐term safety are taken into consideration.

To test potential inducers of HbF, a sensitive and specific reporter assay is required. To this end, Breveglieri et al. generated K562 cells carrying fluorescent reporters under the control of the fetal and adult β‐like globin genes.^14^ Their 2019 paper provides an overview of previously published reporter systems to discover novel inducers of HbF.^14^ These assays rely on reporter constructs in BAC, YAC, or plasmid DNA in different cellular backgrounds including human (K562) and mouse (MEL, GM979) erythroid leukemia cell lines.^15^, ^16^, ^17^, ^18^ None of these reporters tagged the endogenous fetal globin genes *HBG1* or *HBG2* (encoding Aγ and Gγ globin chains, respectively). Tagging the endogenous *HBG* genes would report their expression in a native chromatin context. We hypothesized that this would provide an assay with high specificity and sensitivity when screening for activators of HbF expression. Furthermore, none of the previous HbF reporter systems capitalized on HUDEP2 cells, a human erythroid progenitor cell line that is currently widely used to study the hemoglobin switch.^19^ A major advantage of HUDEP2 cells is that they closely resemble human adult erythroid progenitors and that they do not increase HbF expression under stress conditions, a phenomenon observed with primary erythroid progenitors.^20^ Alternatively, phenotypic screens can be conducted with enzyme‐linked immunosorbent assay (ELISA)‐based methods, as previously performed in primary erythroid cells^21^ and HUDEP2 cells.^22^ However, for technical reasons, these studies were limited to screening fewer than 20,000 compounds. Performing high‐throughput screening (HTS) will increase the likelihood of identifying candidate drugs successfully. HTS typically screens over 100,000 compounds and requires a robust, sensitive, and highly specific assay. An HbF reporter cell line would allow unbiased drug screens to identify potential inducers active in any of the pathways regulating HbF levels in adult red blood cells. To our knowledge, a suitable cell‐based assay reporting endogenous expression of HbF is not yet available. Here, we describe the generation and validation of such a reporter cell line that is compatible with HTS. The cell line reduces time and costs per tested molecule in the search for novel inducers. It also presents an easy‐to‐use benchtop assay to optimize conditions for (epi)genetic activation of HbF expression.

## MATERIALS AND METHODS

### Human erythroid cells

The HUDEP human erythroid cell lines have been described elsewhere.^19^ Cultures of primary human erythroid progenitors^20^, ^23^, ^24^, ^25^ were established from anonymized buffy coats (Dutch blood bank Sanquin, project NVT0146) or ~10 mL of peripheral blood derived from SCD patients (Erasmus MC MEC‐2018‐1422).

### Cell culture

Cells were cultured at 5% CO_2_, 20% O_2_, and 95% ± 5% humidity at 37°C. Cell density was kept between 0.3 and 1.5 × 10^6^ cells/mL refreshing the medium every second or third day. The cells were cultured in fully defined serum‐free Cellquin medium developed at Sanquin Research.^20^, ^23^, ^24^ Cellquin medium was prepared on site from Iscove's modified Dulbecco's medium (IMDM) (Cat. #P04‐20 250; PAN Biotech); the full list of ingredients is provided in Table 1. A readily prepared version of Cellquin is available (Cat. #P04‐20251K; PAN Biotech). Doxycycline, used in the proliferation medium, promotes the expression of the HPV16‐E6/E7 transgene that was introduced to immortalize the HUDEP cells^19^ and was added to the cultures every other day. In the differentiation medium, doxycycline was removed to allow the cells to exit the cell cycle, which is required for terminal differentiation. Cell density and diameter distribution of HUDEP cultures were determined with a CASY Model TTT electrical current exclusion cell counter (Omni Life Science). For storage in liquid nitrogen, HUDEP cells were frozen in fetal calf serum (FCS, Cat. #FBS‐12A; Capricorn Scientific) containing 10% dimethyl sulfoxide (DMSO), typically storing 5–20 million cells in 1 mL suspension.

**Table 1:** IMDM‐based Cellquin cell culture medium.

Additive	Final concentration	Supplier	Cat. Number
For base medium			
Poly(vinyl alcohol) (PVA)	1 g/L	Sigma‐Aldrich, Munich, DE	363081
Human holotransferrin	300 mg/L	Sanquin, NL via Mebiopharm, Tokyo, JP	NA
Cholesterol	2.5 mg/L	Sigma‐Aldrich, Munich, DE	C3045
l‐a‐phosphatydilcholine	2.5 mg/L	Sigma‐Aldrich, Munich, DE	P3556
Oleic acid	1.5 mg/L	Sigma‐Aldrich, Munich, DE	O1383
Insulin	10 mg/L	Sigma‐Aldrich, Munich, DE	I9278
l‐Glutamine	2 mM	PAN Biotech, Aidenbach, DE	P04‐80100
Sodium pyruvate	100 μM	Thermo Fisher Scientific, Waltham, MA	11360070
Penicillin/	50,000 U/L	Sigma‐Aldrich, Munich, DE	P0781
Streptomycin	50 mg/L		
For proliferation medium			
Epoetin alfa	2000 U/L	Janssen‐Cilag, Breda, NL	NA
Human recombinant stem cell factor	100 µg/L	ITK Diagnostics, Uithoorn, NL	K0921139
Dexamethasone	0.4 mg/L	Sigma‐Aldrich, Munich, DE	D4902
Doxycycline	1 mg/L	Sigma‐Aldrich, Munich, DE	D9891
For differentiation medium			
Epoetin alfa	10,000 U/L	Janssen‐Cilag, Breda, NL	NA
Human plasma	3% (v/v)	Sanquin, Amsterdam, NL	NA
Heparin	3125 U/L	STEMCELL Technologies, Vancouver, CA	07980

HEK293T cells were cultured at 5% CO_2_, 20% O_2_, and 95% ± 5% humidity at 37°C in DMEM supplemented with 50,000 U/L penicillin, 50 mg/L streptomycin (Cat. #P0781; Sigma‐Aldrich), and 10% FCS (Capricorn Scientific). Cell density was kept below 90% confluence.

### Sanger sequencing

Polymerase chain reaction (PCR)‐amplified DNA fragments (Supporting Information S1: Table S1) were subjected to Sanger sequencing and analyzed using CLC Main Workbench 8 (Qiagen). To estimate Cas9 efficiency, Sanger sequence trace deconvolution was performed with TIDE.^26^

### Guide RNA (gRNA) design

CRISPOR^27^ was used to design gRNAs in the 3′‐UTR of *HBG1*. Two *HBG1*‐specific gRNAs were identified (Supporting Information S1: Table S1). Template DNA for homology‐directed repair was derived from plasmid DNA or synthesized as a single‐stranded oligonucleotide (Supporting Information S1: Table S1; IDT).

### Nucleofection

HUDEP2 cells (2 × 10^6^) were nucleofected (EW‐113 Program 1) on a 4D‐Nucleofector system (Lonza) using pLentiCRISPR v2 (Addgene #52 961)^28^ plus double‐stranded template DNA or Cas9‐sgRNA ribonucleoprotein (RNP) complex plus single‐stranded template DNA (Alt‐R CRISPR‐Cas9 system; IDT). To isolate clones, cells were single‐cell sorted (FACSARIA III; Becton Dickinson) 2 days postnucleofection.

### Aγ‐HiBiT detection

The Nano‐Glo HiBiT lytic assay (#N3030; Promega) was used for Aγ‐HiBiT luminescence detection with a GloMax plate reader (Promega). Western blots were probed using the Nano‐Glo HiBiT blotting assay (#N2410; Promega).

### SNP arrays

HUDEP2 DNA was used for the Global Screening Array (GSAv3); data were analyzed with GenomeStudio (Illumina).

### Next generation sequencing

RNAseq and ATACseq were performed as described.^29^ Reads were mapped against the GRCh38 human reference using HiSat2 (version 2.1.0).^30^ RNA expression values were called using htseq‐count.^31^

### Flow cytometry

CD253a (#561775; Becton Dickinson) and CD117 (#562435) were used for flow cytometry (LSR‐FORTESSA; Becton Dickinson); data were analyzed with Flowjo v.10.6 (Becton Dickinson).

### Quantitative PCR

Globin expression was determined by qPCR using PSMD1 as a reference (Supporting Information S1: Table S1).^29^, ^32^, ^33^ The $2^{-∆∆C_{t}}$ method was used to calculate expression levels.^34^

### Protein analysis

High‐performance liquid chromatography (HPLC) analysis of HbF/HbA and western blots were performed as described.^29^ Primary antibodies recognized γ‐globin (sc‐21756; Santa Cruz Biotechnology), β‐globin (sc‐21757), and NPM1 (ab10530; Abcam). Appropriate secondary antibodies enabled detection with an Odyssey CLx Imaging System (LI‐COR Biosciences).

### HTS

Assay optimization for the use of Aγ‐HiBiT cells in HTS applications and the pilot screen of 5632 known bioactive drugs were performed at the EMBL Chemical Biology Core facility.

### Statistical tests

Statistical analyses were performed using two‐tailed *t*‐tests (GraphPad Software). *p < 0.05; **p < 0.01; ***p < 0.001.

## RESULTS

### Sequence analysis for *HBG1*‐specific CRISPR‐Cas9 guide RNA designs

**Figure 1:**
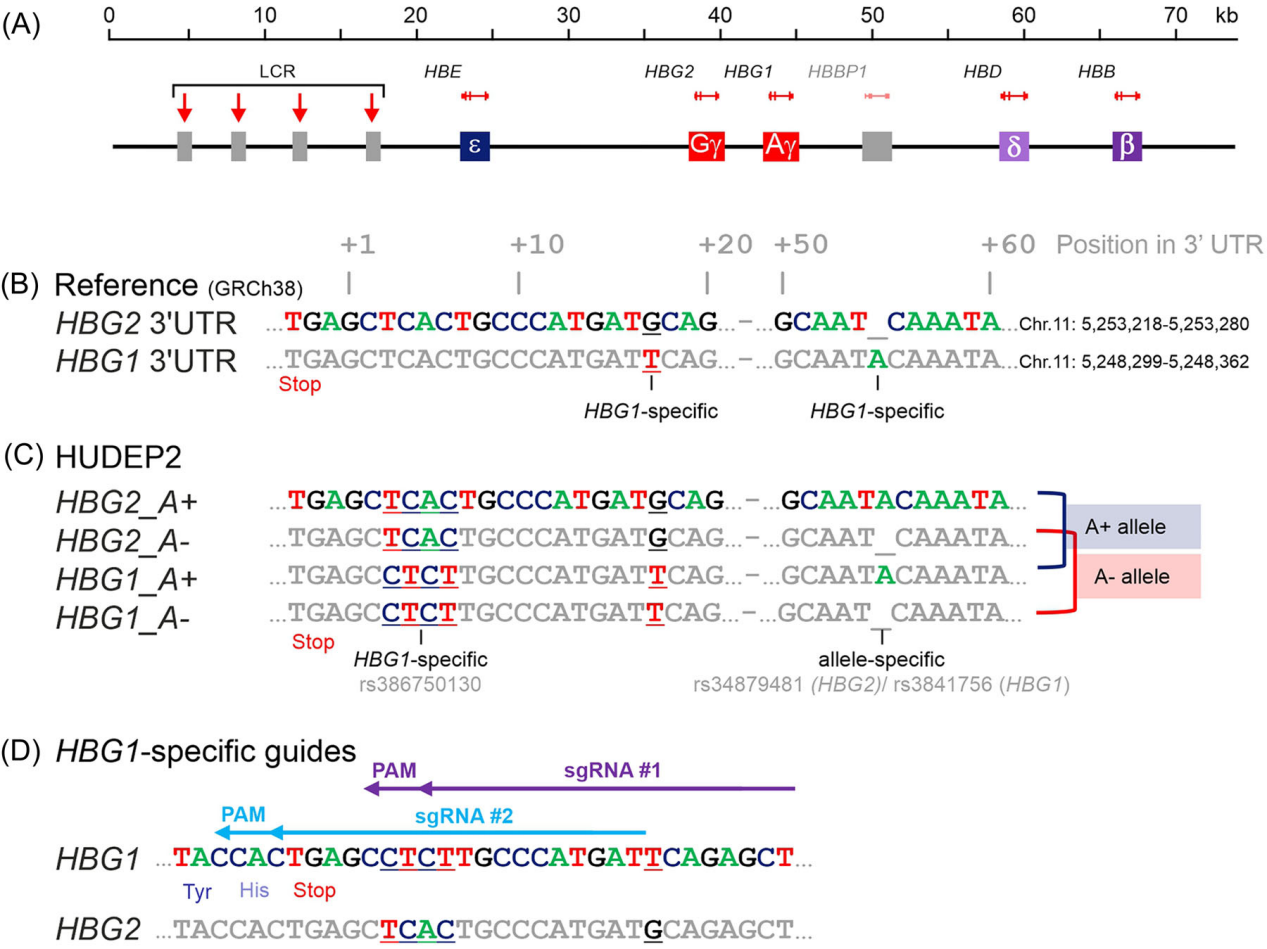
Design of gRNAs specifically targeting the *HBG1* 3′‐UTR in HUDEP2 cells. (A) The human *HBB* locus on chromosome 11 contains 5 β‐like globin genes, arranged in developmental order of expression, and an upstream locus control region (LCR; red arrows indicate DNaseI hypersensitive sites). (B) Comparison of the *HBG1/2* 3′‐UTR sequences from the GRCh38 reference genome identified two *HBG1*‐specific nucleotides at positions +17 and +55 downstream of the TGA stop codon. (C) In HUDEP2 DNA, additional gene‐specific nucleotides were identified. The +55 adenosine is allele‐specific rather than gene‐specific. (D) Two gRNAs designed to specifically target the *HBG1* 3′‐UTR.

The fetal γ‐globin chains are encoded by two nearly identical and closely spaced genes, *HBG1* and *HBG2*. The high level of sequence homology between the two genes poses the risk of simultaneous Cas9‐induced double‐strand breaks at both genes resulting in large deletions or inversions.^35^ CRISPR‐Cas9‐mediated tagging of the endogenous fetal γ‐globin chains therefore requires a gene‐specific gRNA design that leaves the other β‐like globin genes in the *HBB* locus unaffected (Figure 1A,B). A comparison of the two 3′‐UTR regions in the human reference genome GRCh38 identified a unique nucleotide at position +17 (G_*HBG2*_ vs. T_*HBG1*_). Also, *HBG1* contains an extra A at position +55 that is not present in *HBG2* (Figure 1B). In HUDEP2 cells, gene‐specific PCR amplicons of *HBG1* and *HBG2* were sequenced, identifying four unique nucleotides in the 3′‐UTR between positions +3 and +6 (TCAC>CTCT, a polymorphism known with variant ID: rs386750130) (Figure 1C). Of note, this 4‐nucleotide variant was also found in the *HBG1* 3′‐UTR of HUDEP1 and HEK293T cells. Furthermore, in HUDEP2 cells, the extra A at position +55 was allele‐specific rather than gene‐specific. Sanger sequencing of cloned 5 kb amplicons that contained the 3′‐UTRs of *HBG1* and *HBG2* showed that on one chromosome, both genes contain the extra A (A+ allele), while on the other chromosome, both genes lack the +55A (A‐ allele) (Figure 1C). This allele‐specific variant allows differentiation between sequence data from either allele when evaluating the knockin of a reporter gene. These +55A variants are listed in dbSNP as rs3841756 (*HBG1*) and rs34879481 (*HBG2*). With this sequence information, two gRNAs specifically targeting the *HBG1* 3′‐UTR were designed. gRNA #1 maps to an *HBG1*‐specific PAM (protospacer adjacent motif, 5′‐NGG‐3′) and contains 3 gene‐specific nucleotides in the spacer sequence, while gRNA #2 contains 4 gene‐specific nucleotides and directs Cas9 to cut at the Aγ‐globin stop codon (Figure 1D).

### Tagging the endogenous *HBG1* gene in HUDEP cells

**Figure 2:**
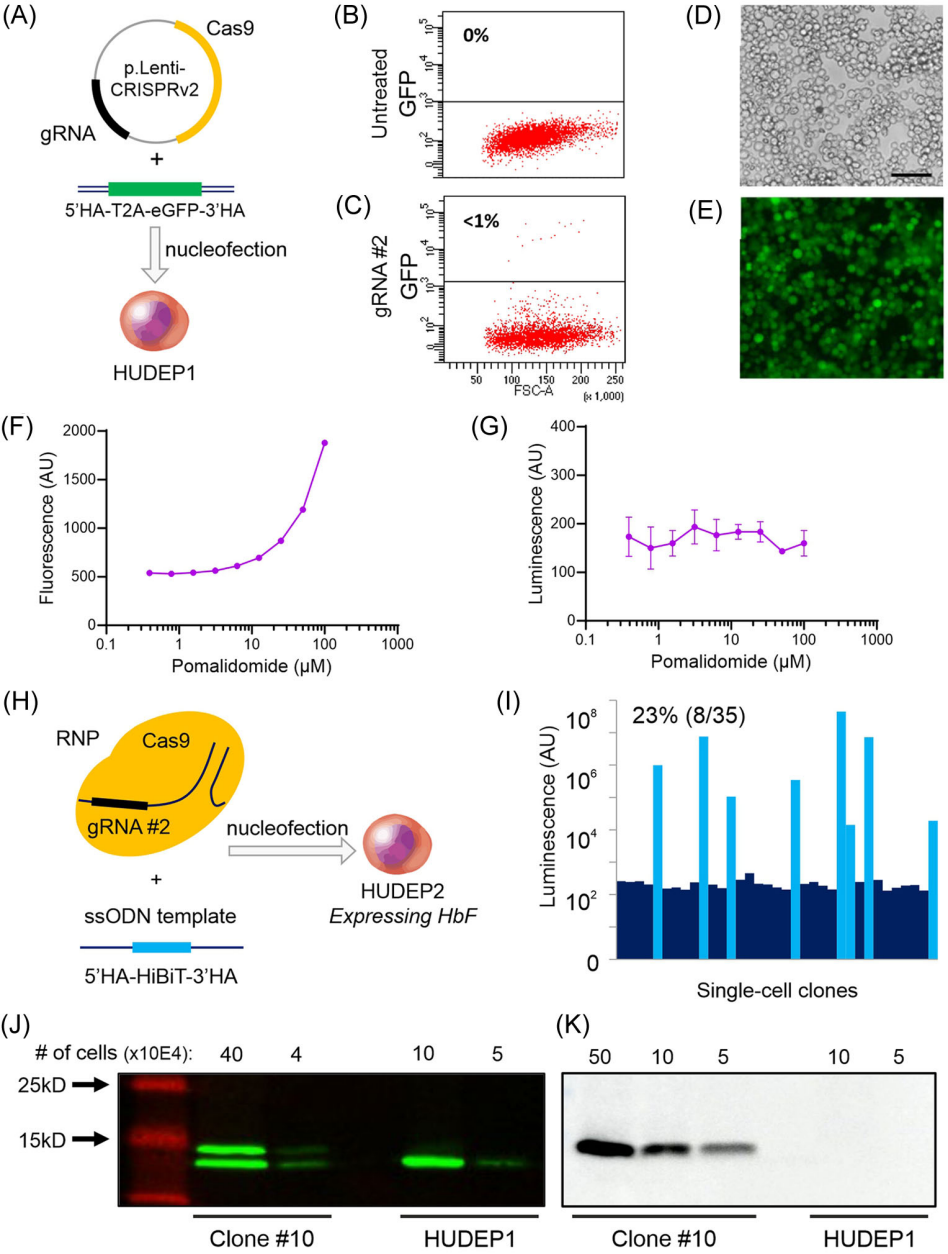
C‐terminal tagging of the *HBG1* gene. (A) Schematic approach of tagging *HBG1* with a GFP reporter gene in the fetal‐like HUDEP1 cells. (B, C) Since HUDEP1 cells express HbF, cells expressing Aγ‐GFP can be enriched by FACS for sequence analysis confirming the successful tagging of *HBG1*. Tagging of *HBG1* is possible albeit at low (<1%) efficiency. (D, E) GFP‐sorted cells under phase contrast and fluorescence microscope. Scale bar = 50 µm. (F) Fluorescence read‐out on a range of pomalidomide concentrations in culture medium showed compound auto‐fluorescence. (G) Luminescence read‐out was not affected by the auto‐fluorescence of pomalidomide. (H) Schematic representation of the tagging strategy with the HiBiT tag, which was optimized in HUDEP2 cells displaying high HbF expression. (I) Use of a single‐strand DNA template and improved delivery method (RNP instead of plasmid‐based) increased the knockin efficiency. Twenty‐three percent (8/35) of single‐cell clones expanded after nucleofection showed an increased luminescent signal. (J) The γ‐globin monoclonal antibody detected an additional slower‐migrating band in the homozygously tagged Clone #10. (K) Only the slower‐migrating band was detected when the western blot was probed for a HiBiT luminescent signal.

The *HBG1*‐specific gRNAs were cloned into an expression plasmid containing Cas9 (p.LentiCRISPRv2 Addgene #52961^28^) and transfected into HEK293T cells to evaluate their DNA cutting efficiency. Sequence trace deconvolution with the TIDE algorithm^26^ suggested gRNA #2 was most efficient (1 bp insertion in 12.9% of traces vs. 5.7% for gRNA #1). *HBG1* was then targeted in the fetal‐like HUDEP1 cells^19^ to test the feasibility of CRISPR‐Cas9‐mediated tagging of *HBG1*. The Cas9_gRNA #2 plasmid was combined with a double‐strand DNA template containing eGFP flanked by 800 bp homology arms (HA, Figure 2A). Although the knockin efficiency was low, indicated by less than 1% GFP+ cells in flow cytometry (Figure 2B,C), the correctly edited cells could be enriched by fluorescence‐activated cell sorting (FACS) (Figure 2D,E). DNA sequence analysis of the sorted cells confirmed that the tag landed correctly in the endogenous *HBG1* gene (data not shown). For a useful reporter cell that could identify novel inducers of HbF expression, *HBG1* must be tagged in HUDEP2 cells that do not express HbF. Without expression of the tagged gene, FACS enrichment of the correctly edited cells would be impossible underscoring the need for a more efficient knock‐in strategy. Also, GFP was not considered the optimal reporter gene. Detection of fluorescence using microscopy or flow cytometry would pose significant limitations in terms of sensitivity and sample analysis time, and drug screens would be hampered by the inherent auto‐fluorescence of some of the pharmacological compounds, for example, pomalidomide (Figure 2F). In contrast, luciferase‐based assays are amenable to fast, specific, and sensitive robotized analysis and are not affected by auto‐fluorescence (Figure 2G). We opted for a split NanoLuciferase system^36^ in which an 11‐amino acid tag (HiBiT, **Hi**gh‐affinity small **BiT**) is fused in‐frame to *HBG1*. Upon lysis of the cells, its counterpart **L**ar**g**e **BiT** (LgBiT) is added to form catalytically active luciferase. The luminescent signal intensity provides a direct and sensitive measure for the amount of Aγ‐HiBiT protein in the lysate. To optimize editing efficiency, we used a HUDEP2 population in which expression of the *HBG1/2* genes had been spontaneously reactivated (TV and SP, unpublished results). First, Cas9 cutting efficiency was improved by delivering Cas9 and the gRNA as a ribonucleic protein (RNP) complex rather than on an expression plasmid (Figure 2H). Second, a single‐strand oligodeoxynucleotide (ssODN) template was used to introduce the HiBiT tag in‐frame at the C‐terminus of Aγ‐globin (Figure 2H). Combined, the knockin efficiency increased to over 20% (Figure 2I). Sequence analysis demonstrated correct knockin of the HiBiT‐tag at the *HBG1* gene. This was confirmed by western blot analysis of Clone #10 in which both *HBG1* genes contain the HiBiT tag. Compared to the untagged Gγ‐chains, the Aγ‐HiBiT chains were detected as a slower migrating band by the γ‐globin antibody (Figure 2J). Importantly, when the HiBiT luminescence assay was used to probe the western blots, only the slower migrating Aγ‐HiBiT band was detected (Figure 2K). To generate the reporter cell line, this procedure was repeated in HUDEP2 cells that did not express HbF. Here, we report the clonal isolation, molecular characterization, and HTS validation of the HbF reporter cell line.

### The Aγ‐HiBiT reporter cell line resembles HUDEP2 cells in proliferation and differentiation

**Figure 3:**
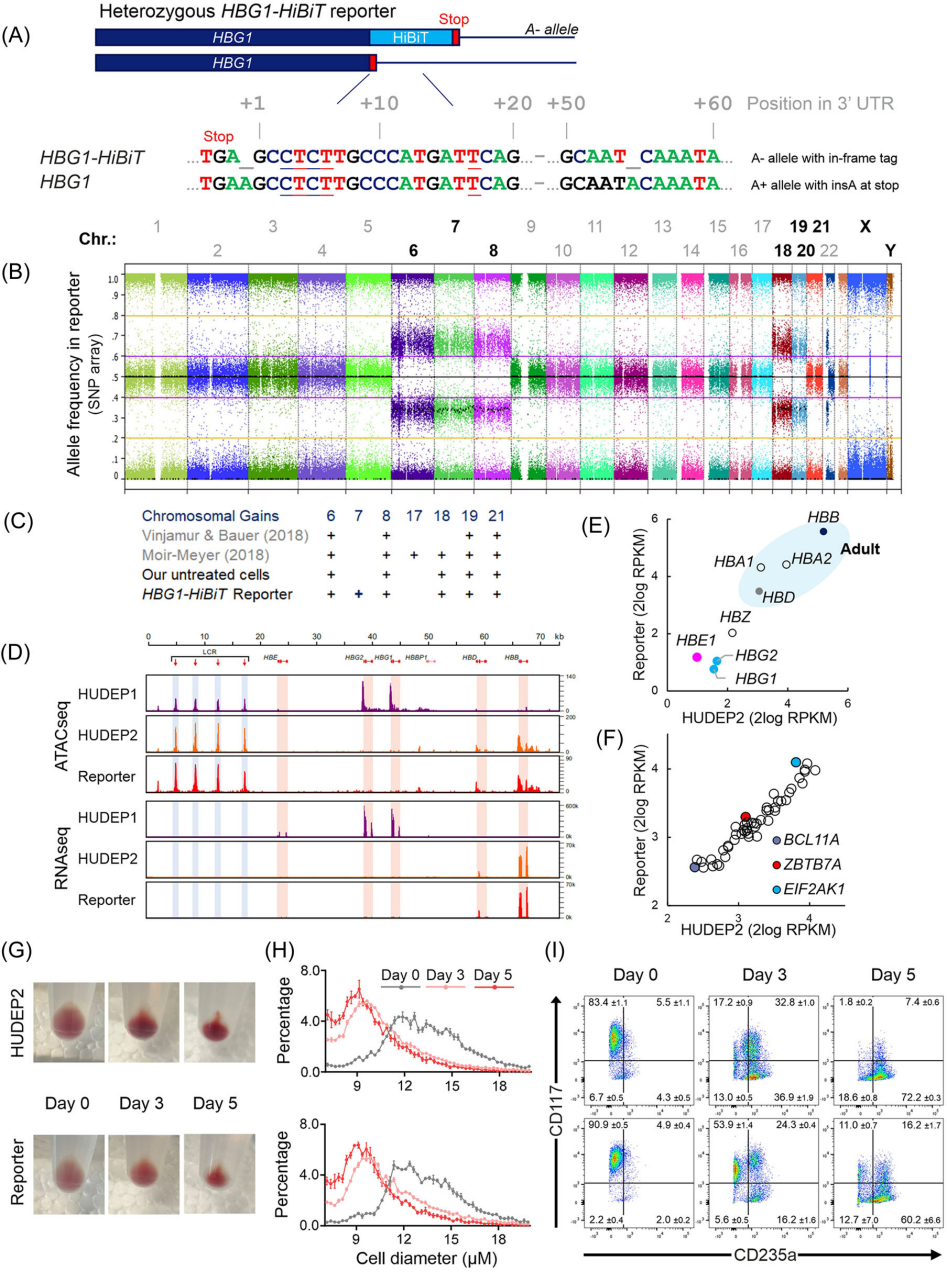
Characterization of the Aγ‐HiBiT reporter cell line. (A) The Aγ‐HiBiT reporter cell line carries a HiBiT tag at the C‐terminus of *HBG1* on the A‐ allele. (B, C) After genome editing and single‐cell cloning, the Aγ‐HiBiT reporter cell line contained an extra chromosome 7 in addition to known HUDEP2 trisomies. (D) ATACseq and RNAseq traces of the *HBB* locus*.* (E) RNA expression levels of hemoglobin subunits in Aγ‐HiBiT reporter and control HUDEP2 cells (RPKM = Reads Per Kilobase per Million mapped reads). (F) RNA expression levels of known HbF regulators in Aγ‐HiBiT reporter and control HUDEP2 cells. (G) HUDEP2 and Aγ‐HiBiT reporter cell pellets before (Day 0) and after differentiation (Day 3 and Day 5). (H) Cell diameter of HUDEP2 and Aγ‐HiBiT reporter cells decreased upon differentiation. *N* = 3 independent cultures, error bars indicate standard error of the mean. (I) During differentiation of HUDEP2 and Aγ‐HiBiT reporter cells, CD117 expression was lost while CD235a expression increased. Percentages in each quadrant are the average of *N* = 3 independent cultures, plus/minus standard deviation.

Sequence analysis confirmed that the clonal Aγ‐HiBiT reporter cell line contained an in‐frame knockin of the HiBiT‐tag at the C‐terminus of *HBG1* on the A− allele. On the A+ allele, an insA was observed in the stop codon, leaving the TGA stop codon intact (Figure 3A). This insertion probably prevented further cutting by Cas9 resulting in heterozygous rather than homozygous knockin of the HiBiT‐tag. Since the sequence of the *HBG2* genes was unaffected (data not shown), 1 out of 4 *HBG* genes now carried the HiBiT‐tag. After genotyping this correctly tagged cell line, its phenotype was characterized and compared to untreated HUDEP2 cells. Untreated HUDEP2 cells are known to have multiple chromosomal gains. Trisomies have been described for chromosomes 6, 8, 17, 18, 19, and 21.^37^, ^38^ In line with these reports, SNP array analysis of our untreated HUDEP2 cells revealed trisomy for chromosomes 6, 8, 18, 19, and 21. The reporter clone contained an additional copy of chromosome 7, but no chromosomes were lost (Figure 3B,C). A comparison of open chromatin in the *HBB* locus of HUDEP1, HUDEP2, and the reporter cells with ATACseq showed that the ATAC sites in the reporter cells matched the pattern observed in HUDEP2 cells (Figure 3D). The absence of ATACseq peaks at the *HBE*, *HBG1,* and *HBG2* genes indicated that these genes remained effectively silenced. RNA sequence traces confirmed that transcriptional activity in the reporter cells is limited to the adult *HBD* and *HBB* genes (Figure 3D,E). Expression levels of 52 previously reported regulators of HbF were compared between the reporter cells and control HUDEP2 cells (Supporting Information S1: Table S2). The expression profile of these HbF‐regulating factors, including the two key repressors of HbF, *BCL11A* ^39^ and *ZBTB7A,* ^40^ correlated closely between the two cell lines (Figure 3F). The *EIF2AK1* gene, encoding a kinase and known repressor of HbF,^41^ is located on chromosome 7. The reporter cell line, with three copies of chromosome 7, showed ~2‐fold increased expression of this gene. Two other reported HbF regulators located on chromosome 7, *JAZF1* ^42^, ^43^ and *EZH2,* ^44^, ^45^ were not upregulated in the reporter cell line. The expression pattern of hemoglobin subunits in the reporter cell line closely resembles that observed in HUDEP2 cells. Both lines express very low levels of HbF and high levels of HbA. Hemoglobin expression depends on the differentiation state of the culture at the time of RNA isolation, which might explain the minor differences in the absolute number of transcripts picked up by RNAseq (Figure 3E). Upon induction of differentiation, HUDEP2 cell diameter decreases, while the expression of globin chains gradually increases. The parental HUDEP2 and reporter cells responded similarly after the medium was changed to differentiation conditions. Upon differentiation, the red color of the cell pellets increased indicating higher levels of globin expression, while cell diameter decreased (Figure 3G,H). Flow cytometry showed that during differentiation the reporter cells changed expression of cell surface markers similar to differentiating HUDEP2 cells, that is, loss of CD117 and gain of CD235a (Figure 3I). Together these results confirmed that the Aγ‐HiBiT reporter cell line closely resembles the adult erythroid progenitor phenotype of HUDEP2 cells.

### Application of the Aγ‐HiBiT reporter cell line to evaluate genetic HbF‐inducing strategies

**Figure 4:**
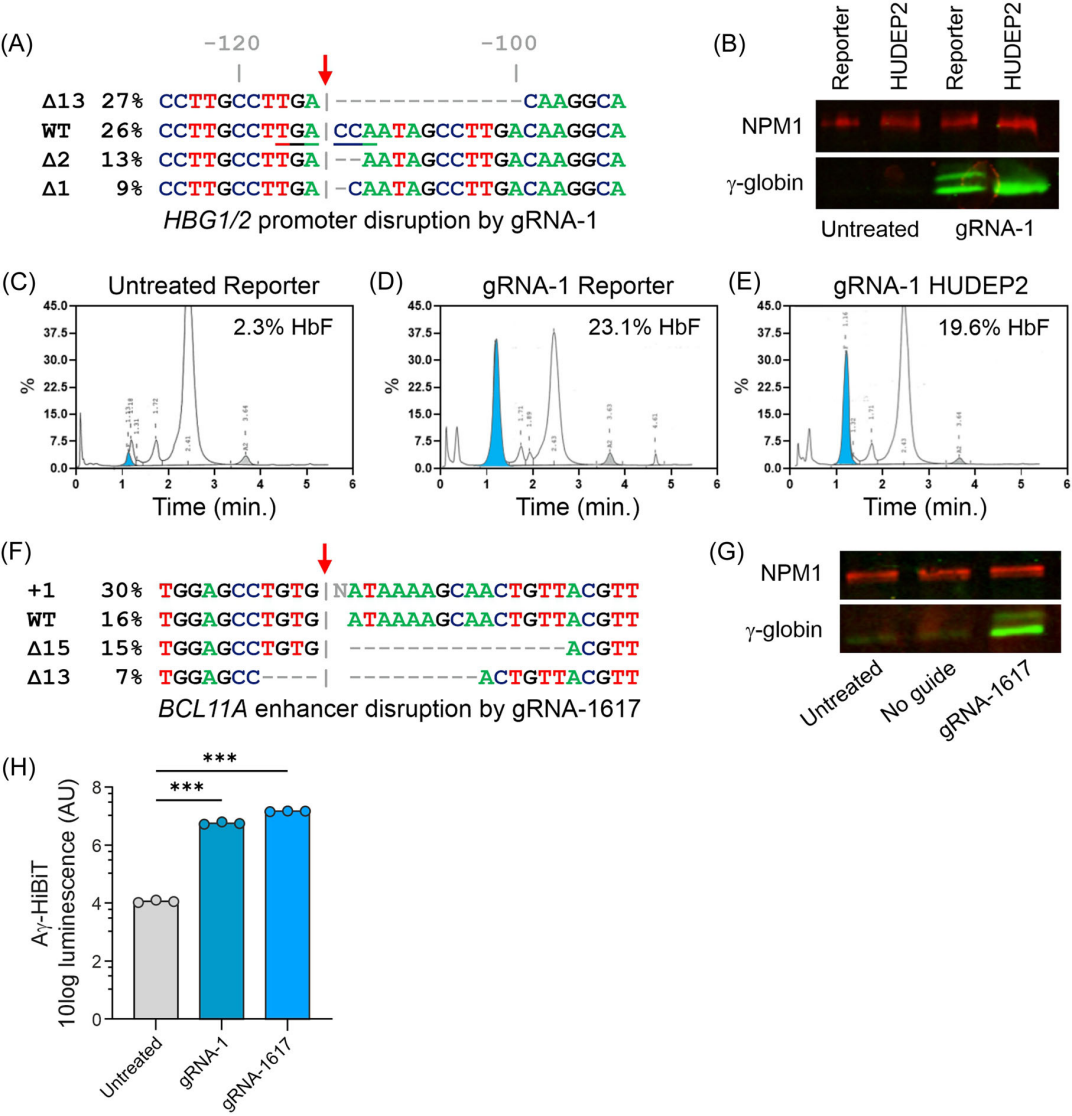
Use of Aγ‐HiBiT reporter cells to evaluate genetic fetal hemoglobin (HbF)‐induction. (A) Deconvolution of Sanger sequence traces showed Cas9‐mediated disruption of a BCL11A binding site (TGACCA) in the *HBG1/2* promoter of Aγ‐HiBiT reporter cells. Similar results were obtained in control HUDEP2 cells. The red arrow indicates the predicted Cas9 cleavage position relative to the transcription start site. (B) On western blot, the γ‐globin antibody detected *HBG1/2* induction after promoter disruption of Aγ‐HiBiT reporter and control HUDEP2 cells. NPM1 served as loading control. (C–E) High‐performance liquid chromatography detected an increase in HbF tetramers after genetic editing of the Aγ‐HiBiT reporter cells similar to the increase observed in edited control HUDEP2 cells. (F) Deconvolution of Sanger sequence traces showed Cas9‐mediated disruption of an enhancer of HbF‐repressor *BCL11A* in Aγ‐HiBiT reporter cells. The red arrow indicates the predicted cleavage position. (G) On western blot, the γ‐globin antibody detected *HBG1/2* induction after *BCL11A* enhancer disruption of the Aγ‐HiBiT reporter cells. (H) The HiBiT Lytic assay reported Aγ‐HiBiT induction after the Aγ‐HiBiT reporter cells had been exposed to the two orthogonal genetic approaches. Note the logarithmic scale (10 log).

In genetic experiments, the Aγ‐HiBiT reporter cell line was evaluated as a tool to quickly evaluate different genetic HbF‐inducing strategies. This requires that the reporter cells survive gene editing experiments. Also, any increase in detected Aγ‐HiBiT signal should correlate quantitatively with the extent of HbF induction as measured by standard methods, such as western blotting or HPLC. Using Cas9 guided to the promoters of *HBG1* and *HBG2* by a gRNA previously published by Traxler et al.,^46^ a 13 bp deletion is preferentially introduced. This deletion is a known HPFH mutation.^47^ In the reporter cells, the HPFH mutation was successfully introduced in ~27% (estimate based on sequence trace deconvolution) of the *HBG1/2* promoters (Figure 4A). After editing the *HBG1/2* promoters in HUDEP2 and the reporter cells, γ‐globin protein levels increased in both cell types (Figure 4B). Western blots on lysates from the reporter cells demonstrated induction of HiBiT‐tagged and untagged γ‐globin chains. The lower intensity of the slower migrating Aγ‐HiBiT chain fits with the notion that the HiBiT tag was introduced in one of the four *HBG* genes (Figures 3A and 4B). Untreated reporter cells contained 2.3% HbF, which increased to 23.1% in the population after gene editing, as was quantified by HPLC (Figure 4C,D). Similarly, in the HUDEP2 population, the CRISPR experiment increased the HbF levels to 19.6% (Figure 4E). As an alternative genetic approach, the reporter cells were subjected to editing outside the *HBB* locus with the disruption of an erythroid‐specific enhancer of *BCL11A,* a key repressor of HbF.^48^ Also, for this orthogonal strategy, sequence disruption and HbF induction were observed (Figure 4F,G). Next, we tested if the induction of HbF was reflected by the luminescent reporter signal. The HiBiT Lytic Assay showed a sharp increase in luminescence signal from the reporter cells after gene editing of the *HBG* promoters (gRNA‐1, Figure 4H) and the *BCL11A* enhancer (gRNA‐1617, Figure 4H).

### Pomalidomide as a positive control compound for HbF induction

**Figure 5:**
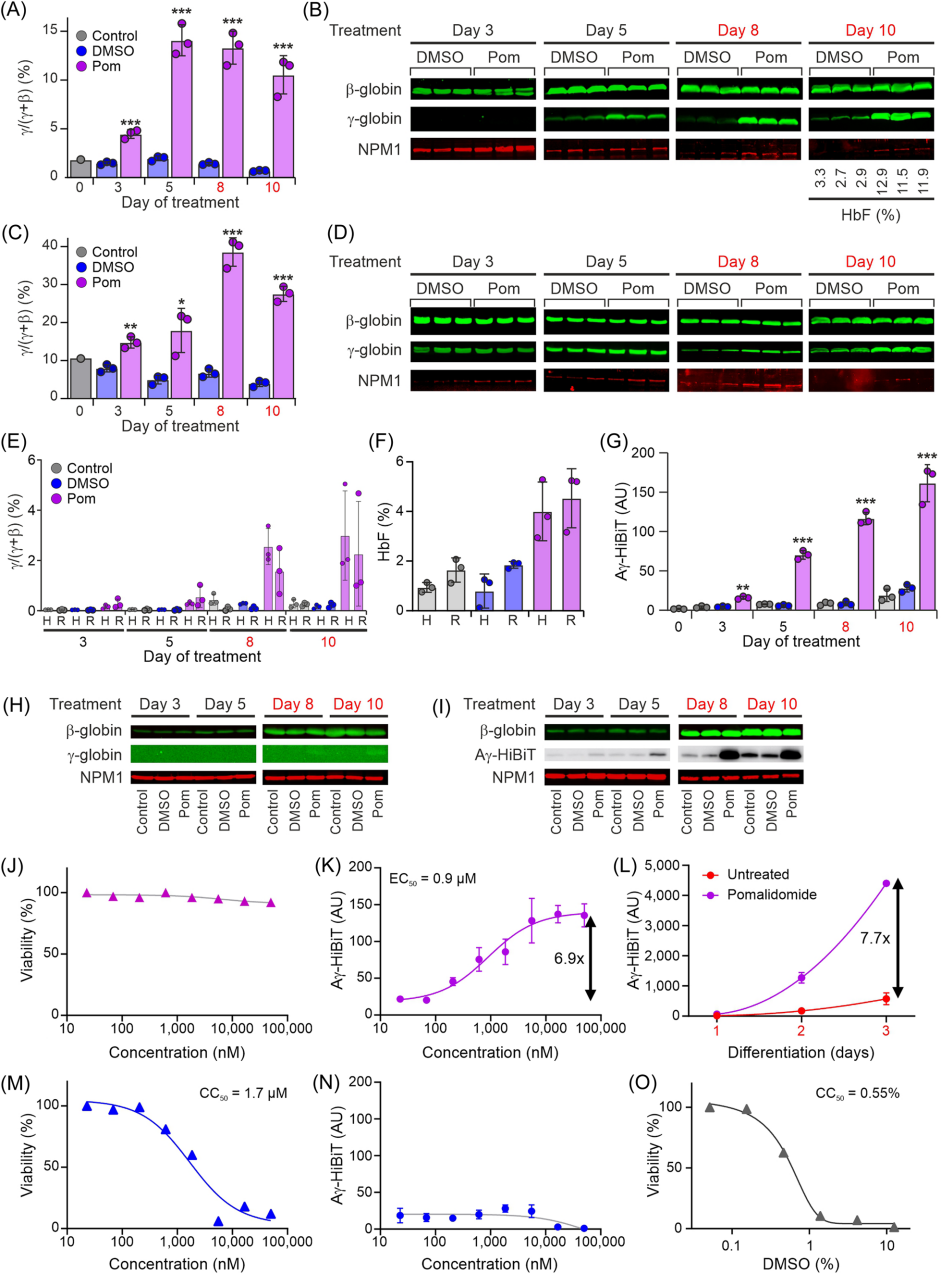
Use of pomalidomide as a positive control for fetal hemoglobin (HbF) induction. (A) Primary erythroid progenitors derived from a healthy donor were treated with 10 μM pomalidomide under proliferation conditions for 5 days and then switched to differentiation conditions. Samples for quantitative reverse‐transcription polymerase chain reaction analysis of γ‐globin expression were taken at Days 0, 3, 5 (proliferation conditions) and Days 8 and 10 (differentiation conditions, red font). Treatment: gray: control; blue: dimethyl sulfoxide (DMSO) (solvent control), purple: 10 μM pomalidomide. *N* = 3 independent cultures; error bars indicate standard deviations. (B) Western blot analysis of β‐globin and γ‐globin expression of DMSO and pomalidomide (Pom) treated samples shown in (A) HbF levels were measured by high‐performance liquid chromatography (HPLC) at Day 10 of culture. (C) Primary erythroid progenitors derived from an sickle cell disease patient were treated with 10 μM pomalidomide under proliferation conditions for 5 days and then switched to differentiation conditions. Other details are as for panel A. (D) Western blot analysis of β‐globin and γ‐globin expression of DMSO and pomalidomide (Pom) treated samples shown in (C). HbF levels could not be measured by HPLC at Day 10 of culture due to insufficient number of cells. (E) HUDEP2 and Aγ‐HiBiT reporter cells were treated with 10 μM pomalidomide under proliferation conditions for 5 days and then switched to differentiation conditions. H = HUDEP2 cells; R = Aγ‐HiBiT reporter cells. Other details are as for panel A. (F) HbF levels were measured by HPLC at Day 10 of culture of HUDEP2 cells and Aγ‐HiBiT reporter cells. See panel (E) for other details. (G) The HiBiT lytic assay reported Aγ‐HiBiT induction after the Aγ‐HiBiT reporter cells had been exposed to 10 μM pomalidomide (see E, F). (H) Western blot analysis of β‐globin and γ‐globin expression of control, DMSO, and pomalidomide (Pom)‐treated HUDEP2 cells (see E, F). (I) Western blot analysis of β‐globin and Aγ‐HiBiT expression of control, DMSO, and pomalidomide (Pom)‐treated Aγ‐HiBiT reporter cells (see E, F). (J, K) Viability and HiBiT lytic assays of Aγ‐HiBiT reporter cells treated for 72 h with pomalidomide at various concentrations showed a maximum 6.9‐fold induction of Aγ‐HiBiT signal (EC_50_ = 0.9 µM), but no toxicity up to 50 µM pomalidomide. Error bars indicate the standard deviation of *N* = 3 independent cultures. (L) Although differentiation alone increased globin expression and thus the Aγ‐HiBiT signal, the addition of pomalidomide to the differentiation medium resulted in an additional 7.7‐fold increase in Aγ‐HiBiT signals. Error bars indicate the standard deviation of *N* = 3 independent cultures. (M, N) A toxic compound (no surviving cells at 50 µM, CC_50_ = 1.7 µM) did not induce Aγ‐HiBiT expression. Error bars indicate the standard deviation of *N* = 3 independent cultures. (O) The Aγ‐HiBiT reporter cells were sensitive to the solvent DMSO (CC_50_ = 0.55% [v/v]).

To use the reporter cell line for the discovery of γ‐globin‐inducing compounds, a compound that can serve as a positive control is required. We used pomalidomide, a thalidomide derivative that was previously described to have HbF‐inducing properties.^49^, ^50^, ^51^, ^52^ Primary erythroid progenitors are known to express relatively high levels of HbF when cultured.^20^ Indeed, we observed a baseline level of ~2% *HBG1/2* as a fraction of *HBG1/2*+*HBB* messenger RNA (mRNA) in primary erythroid progenitors obtained from a healthy donor (Figure 5A). Under proliferation conditions, this increased to 12%–15% at Day 5 of treatment with 10 µM pomalidomide, and this ratio was maintained when pomalidomide treatment was continued under differentiation conditions (Figure 5A). Western blot analysis confirmed these observations on γ‐globin expression upon pomalidomide treatment (Figure 5B). HPLC analysis of cells harvested at the end of the experiment (Day 10) showed that the HbF level of ~3% in untreated cells (DMSO solvent control) had increased to ~12% upon pomalidomide treatment. In primary erythroid cells obtained from an SCD patient, we observed a baseline level of ~10% *HBG1/2* as a fraction of *HBG1/2*+*HBB* mRNA (Figure 5C). This increased to 15%–20% at Day 5 of treatment with 10 µM pomalidomide under proliferation conditions, further increasing to 30%–40% when pomalidomide treatment was continued under differentiation conditions (Figure 5C). Western blot analysis confirmed these observations on γ‐globin expression upon pomalidomide treatment (Figure 5D). In HUDEP2 cells, baseline expression of HbF is low.^19^, ^29^ Following the schedule used for the primary cells (Figure 5A–D), we treated HUDEP2 and the reporter cells with 10 µM pomalidomide and analyzed globin expression by quantitative reverse‐transcription polymerase chain reaction (RT‐qPCR) (Figure 5E). Increased γ‐globin expression was detectable at Day 3 and Day 5, and a further increase was observed when pomalidomide treatment was continued under differentiation conditions, reaching a maximum of 1%–5% at Day 10 (Figure 5E). HPLC analysis of cells harvested at the end of the experiment (Day 10) showed an HbF level of ~1%–2% in untreated cells and of ~3%–5% in pomalidomide‐treated cells (Figure 5F). Detection of Aγ‐HiBiT with the HiBiT Lytic Assay in the reporter cell line showed a significantly increased signal at Day 3 (3.5‐fold, Figure 5G), which increased further until Day 10 (5.8‐fold, Figure 5G). Western blot analysis of the parental HUDEP2 cells showed increased expression of β‐globin during culture under differentiation conditions, while γ‐globin was barely detectable even at Day 10 of pomalidomide treatment (Figure 5H). Similarly, increased expression of β‐globin during culture under differentiation conditions was observed for the reporter cells (Figure 5I), while γ‐globin expression could not be detected with the γ‐globin antibody (data not shown). When the blot was probed with the HiBiT luminescence assay, increased expression of Aγ‐HiBiT was already detectable at Day 3 of pomalidomide treatment and further increased at Days 5–10 (Figure 5I). Collectively, we conclude that HUDEP2 cells and the reporter cell line display very similar patterns of pomalidomide‐mediated induction of γ‐globin. These results also strongly support the specificity and sensitivity of Aγ‐HiBiT detection by the lytic assay (Figure 5G) and on western blots (Figure 5I). Next, we treated reporter cells with a range of pomalidomide concentrations for 3 days in proliferation conditions. ATP quantification as a measurement of cell viability indicated no severe toxicity up to a final concentration of 50 μM pomalidomide (Figure 5J), and we observed a maximum 6.9‐fold increase of Aγ‐HiBiT signal with an EC_50_ around 0.9 µM pomalidomide (Figure 5K). To determine if pomalidomide activates HbF specifically or if it is promoting differentiation with an indirect effect on all globin expression levels, we next tested pomalidomide treatment under differentiation conditions. As expected, the Aγ‐HiBiT signals increased upon induction of differentiation for 3 days. Compared to DMSO‐treated cells, the addition of 50 μM pomalidomide resulted in a further 7.7‐fold increase of the Aγ‐HiBiT signal (Figure 5L). Thus, pomalidomide also increased γ‐globin expression under differentiation conditions. Importantly, treatment with an unrelated compound that displayed high toxicity (CC_50_ = 1.7 µM, Figure 5M) did not increase the Aγ‐HiBiT signal (Figure 5N). This indicated that cytotoxic stress did not reactivate HbF in the reporter cells, an important prerequisite for use in HTS applications. Finally, we tested the sensitivity of the reporter cells to the solvent DMSO. To our surprise, DMSO concentrations above 0.2% affected cell viability (CC_50_ = 0.55%, Figure 5O). For compound screening, final DMSO concentrations should therefore be kept below 0.1%.

### The reporter cell line is HTS compatible

**Figure 6:**
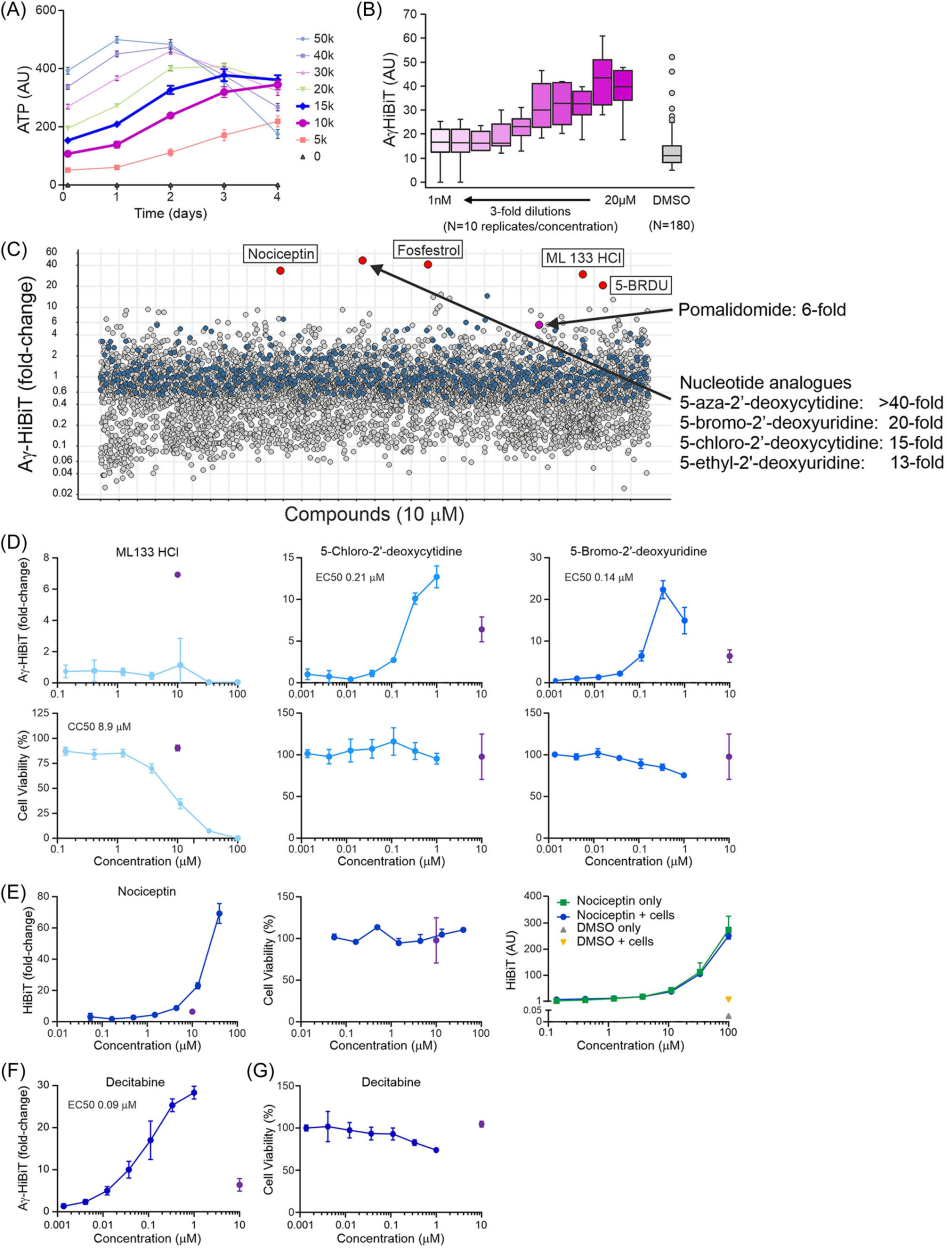
The Aγ‐HiBiT reporter cell line is suitable for high‐throughput screening (HTS) applications aimed at the detection of hemoglobin (HbF) induction. (A) Cell numbers over time measured with viability assay for different cell plating densities in 384‐well plates. (B) Aγ‐HiBiT induction after 3 days of treatment with serial dilutions of pomalidomide in a 384‐well plate starting at 12.5k cells per well. Note the six outliers in the solvent control samples (DMSO). (C) Aγ‐HiBiT induction in a pilot screen of 5632 known medicinal compounds normalized against median DMSO control/plate validated the use of the reporter cell line in HTS assays by identifying the known HbF‐inducer decitabine (5‐aza‐2′‐deoxycytidine) and other nucleotide analogs. (D) Aγ‐HiBiT reporter cells were treated for 3 days with a range of concentrations of the indicated compounds. Top: Aγ‐HiBiT signals normalized to DMSO‐treated cells. Bottom: viability assays performed in parallel. Purple: Treatment for 3 days with 10 µM pomalidomide. *N* = three independent cultures. Error bars indicate standard deviations. (E) Aγ‐HiBiT reporter cells were treated for 3 days with a range of concentrations of nociception. Left: Aγ‐HiBiT signals normalized to DMSO‐treated cells. Middle: Viability assays performed in parallel. Purple: Treatment for 3 days with 10 µM pomalidomide. Right: Nociceptin alone activated luciferase activity in the Nanoluciferase complementation assay. *N* = 3 independent cultures. Error bars indicate standard deviation. (F) Aγ‐HiBiT reporter cells were treated for 3 days with a range of concentrations of decitabine. Aγ‐HiBiT signals were normalized to DMSO‐treated cells. (G) Viability assays performed on cells treated with decitabine (see F). Purple: Treatment for 3 days with 10 µM pomalidomide. *N* = 3 independent cultures. Error bars indicate standard deviation.

Having established that the Aγ‐HiBiT reporter cells resemble the parental HUDEP2 cells and that pomalidomide can serve as a robust positive control for HbF induction, we aimed to validate the assay for high‐throughput drug screening. HTS libraries include up to hundreds of thousands of compounds. To make a large screen feasible and cost‐effective, these compounds are pre‐plated in white opaque 384‐well plates that are specially designed for robotized luminescence assays. The reporter cells would be required to survive plating by a liquid handling robot and a predefined incubation period in the individual wells. Subsequent robotic addition of the HiBiT lytic buffer, which also contains LgBiT and the luciferase substrate, followed by automated luminescence read‐out would enable HTS. The pomalidomide experiments suggested that 3–4 days of incubation are required before measuring HbF induction. Longer incubation times require a medium change, which is undesirable in the HTS setting as this introduces a source of uncontrollable variability. To develop the HTS application, daily viability assays were performed after plating the reporter cells to 384‐well plates in concentrations ranging from 0 to 50,000 cells in 50 µL medium per well. This showed that cultures starting with 10,000–15,000 cells per well displayed a linear expansion for up to 3 days (Figure 6A). Therefore, 12,500 cells per well in 50 µL medium were incubated for 3 days in 384‐well plates pre‐plated with pomalidomide dissolved in DMSO at concentrations ranging from 20 µM to 1 nM (10 concentrations, threefold serial dilutions) (Figure 6B). These initial tests in the 384‐well format showed that further optimization was required. First, the maximum Aγ‐HiBiT induction by pomalidomide, deduced from fully automated luminescence read‐out after the addition of 50 µL lytic buffer per well, was lower than the ~6–7‐fold observed in 96‐well plates. Second, with 0.1% DMSO, some outlier wells occurred (6/180, Figure 6B). Third, with 100 µL total volume per well after the addition of lytic buffer, the airflow created by the automated plate reader caused the transfer of liquid between wells. In addition, high‐speed injection of the lytic buffer created air bubbles. These issues were addressed by adding a reduced volume (25 µL) of lytic buffer at a lower speed and incubating the mixture for 10 min before read‐out by the automated plate reader. The rapid expansion of the reporter cells is another prerequisite for HTS applications. In the fully defined serum‐free proliferation culture medium, the reporter cells expand ~5‐fold every 3 days. We prepared stocks of 20 million cells per vial stored in liquid nitrogen. For large experiments, we typically thaw 60 million cells and plate them at a density of 1 million cells/mL. The cells recover within 24 h after which they can be plated at 0.3 million cells/mL and expanded. Starting with 60 million cells, over 0.5 billion cells can be grown within a week, a number sufficient to perform ~40,000 assays in the 384‐well format.

### A pilot screen of an annotated drug library identifies a known HbF inducer

As a pilot screen, a collection of 5632 known bioactive/medicinal compounds was screened at 10 µM final concentration using the Aγ‐HiBiT reporter cell line (Figure 6C). Pomalidomide was used as a positive control and to normalize for variation between the plates. Compared to the DMSO controls, the majority of the tested compounds resulted in a lower luminescence signal; this was interpreted as a sign of toxicity. The Aγ‐HiBiT signal was detectable in the untreated reporter cells and a decrease in signal, therefore, indicated that the cells failed to survive the 3‐day incubation period. Five compounds (0.089%) showed a >20‐fold induction of the Aγ‐HiBiT signal, while none of the DMSO‐only wells exceeded this threshold (Figure 6C). In addition to the positive control wells, pomalidomide was also present in the screened collection, where it showed the expected sixfold induction. Furthermore, the library contained compounds targeting known HbF regulators. Three inhibitors of histone methyltransferases EHMT1/EHMT2,^53^, ^54^, ^55^, ^56^ UNC0224, UNC0321, and A‐366, displayed 4.9‐, 4.7‐, and 3.8‐fold Aγ‐HiBiT induction, respectively. The histone deacetylase (see Migliaccio et al.^57^ for review) inhibitor Bufexamac showed a 2.7‐fold induction of the Aγ‐HiBiT signal. The modest response to hydroxyurea (1.6‐fold induction) is in agreement with previously reported data.^58^ We then tested serial dilutions of the top hits ML 113 HCl, 5‐chloro‐2′‐deoxycytidine, 5‐bromo‐2′‐deoxyuridine (5‐BRDU), 5‐aza‐2′‐deoxycytidine (Decitabine), and Nociceptin (Fosfestrol was not available for testing). ML 113 HCl did not induce Aγ‐HiBiT expression and affected cell viability (CC_50_ = 8.9 µM, Figure 6D). Strong Aγ‐HiBiT induction was observed upon treatment with 5‐chloro‐2′‐deoxycytidine (EC_50_ = 0.21 µM) and 5‐BRDU (EC_50_ = 0.14 µM); 5‐BRDU displayed a modest impact on cell viability over the concentration range tested (Figure 6D). The addition of Nociceptin, a 17‐amino acid neuropeptide, led to sharply increased signals in the HiBiT Lytic Assay at the high end of the concentration range without affecting cell viability (Figure 6E). Since we found that Nociceptin alone activates the HiBiT Lytic Assay (Figure 6E), we conclude that this is a false‐positive hit. The top hit, with an Aγ‐HiBiT induction over 40‐fold, was decitabine (Figure 6C), a well‐known HbF inducer that has been tested in clinical trials.^50^, ^59^, ^60^ Finding a known activator is a promising outcome of the pilot screen that contributes to the HTS validation of the reporter cell line. Much like typical HTS projects, in this pilot screen, all compounds were tested once at a fixed concentration of 10 μM. In larger screens identifying clusters of active compounds that share similarities in their chemical structures can help prioritize hits that are most likely true positives. In our pilot screen, a cluster of nucleotide analogs including decitabine all showed an induction of Aγ‐HiBiT signal outperforming pomalidomide (Figure 6C,D). Finding such a cluster is promising and helps prioritize hit compounds selected for validation experiments. Treatment of the Aγ‐HiBiT reporter cells with serially diluted decitabine in a 96‐well format confirmed decitabine as a strong HbF‐inducing agent (EC_50_ = 0.09 µM, Figure 6F). Decitabine displayed a modest impact on cell viability over the concentration range tested (Figure 6G). We conclude that the reporter cell line is capable of identifying clusters of HbF‐inducing compounds. Most importantly, the pilot screen validates the Aγ‐HiBiT reporter cell line as a robust tool for the unbiased identification of novel HbF‐inducing compounds using the HTS approach.

## DISCUSSION

Here, we report the development, characterization, and validation of an HbF reporter system in the erythroid progenitor cell line HUDEP2 to speed up the discovery of new treatments for patients with β‐globinopathies. Careful sequence analysis of the human *HBG* genes allowed the design of an *HBG1‐*specific gRNA and CRISPR‐mediated tagging of the *HBG1* gene. In contrast to available HbF reporter cell lines that rely on exogenous DNA constructs, this new reporter cell line contains a short tag at the *HBG1* gene to evaluate endogenous HbF expression. The bioluminescent tagging of Aγ‐globin provides a quantitative and specific readout that is not affected by the auto‐fluorescence of either the cells or the tested compounds. The identification of the known HbF inducer decitabine, out of >5000 compounds tested in the pilot screen, provides confidence in its suitability for high‐throughput drug screening. We provide details of a serum‐free fully defined cell culture medium and recommended cell density and incubation times. The 3‐day incubation period requires compound stability in aqueous solution, possibly explaining why pomalidomide^49^, ^51^, ^52^ (sixfold induction) was active while thalidomide^61^, ^62^, ^63^, ^64^ (1.1‐fold induction) was negative in the pilot screen. Our approach is in line with incubation times of 3–4 days in previously reported ELISA‐based screens in HUDEP2 cells in 384‐well plates.^22^, ^65^ In addition to screening small molecules, the Aγ‐HiBiT reporter cell line provides a bench‐top assay to evaluate genetic HbF‐inducing therapies. Other applications in the pursuit of HbF‐inducing therapies can be tested, for example, for the evaluation of HbF induction by short hairpin RNAs^66^ or microRNAs.^67^, ^68^ The current version of the Aγ‐HiBiT reporter cell line does have several limitations. First, the luciferase assay evaluates the expression of *HBG1* at the population level. For single‐cell analysis (e.g., to determine if HbF induction occurs equally in all cells), we are currently developing a second reporter line that contains a fluorescent tag, making it compatible with flow cytometry analysis. The reported sequence of the 3′‐UTR (that differs from the human reference genome GRCh38) provides a strategy to tag *HBG1*/*HBG2* with any tag of choice. Second, the cells proved to be sensitive to DMSO concentrations above 0.1%, which limits the range of concentrations in which DMSO‐dissolved compounds can be tested. The pilot screen provided proof of concept for HTS testing of compounds at an initial concentration of 10 µM without exceeding the DMSO toxicity limit. For HTS applications, we suggest including a column of 10 μM pomalidomide samples as positive controls on every plate to evaluate plate‐to‐plate variation and to normalize the data of test compounds against the median of the DMSO solvent controls. Lastly, we recommend performing initial screening under proliferation conditions. As shown, starting at the correct cell density allows the expansion of the reporter cells throughout the incubation interval of a compound screen. Screening in proliferating reporter cells is advantageous because fewer cells are required at the plating phase while screening in differentiation conditions would introduce noise arising from variability in differentiation states within and between experiments. Potential limitations of screening in the proliferation state include false‐negative results for HbF inducers that simultaneously block the cell cycle; an increase in the Aγ‐HiBiT signal per cell might be missed if the well contains fewer cells than control wells. Since the HUDEP2‐based Aγ‐HiBiT reporter cells express very low levels of HbF, weak inducers are most at risk of being missed. In addition to false negatives, screening in the proliferation state might also give false positive results if differentiation is induced by the compound tested since during differentiation expression of globins including HbF increases. In rare instances, the compound itself may activate the HiBiT assay. We found that this is the case with Nociceptin, a 17 amino acid peptide. As part of the hit triage strategy, such false positive hits would be quickly identified in validation experiments.

To speed up the discovery of novel HbF‐inducing therapies, laboratories around the world should be working on this pursuit in parallel. The Aγ‐HiBiT reporter cell line is therefore available to the research community through the RIKEN BioResource Research Center Cell Bank, Tsukuba, Ibaraki, JP.

## AUTHOR CONTRIBUTIONS

Thijs C. J. Verheul, Nynke Gillemans, Kerstin Putzker, Rezin Majied, Tingyue Li, Memnia Vasiliou, Bert Eussen, Annelies de Klein, Wilfred F. J. van IJcken, and Ulrike Uhrig performed experiments. Thijs C. J. Verheul, Bert Eussen, Annelies de Klein, Wilfred F. J. van IJcken, and Sjaak Philipsen analyzed data. Emile van den Akker, Marieke von Lindern, and Yukio Nakamura provided essential materials and expertise. Ulrike Uhrig, Joe Lewis, Thamar van Dijk, and Sjaak Philipsen supervised the experiments. Thijs C. J. Verheul, Joe Lewis, and Sjaak Philipsen designed the experiments. Thijs C. J. Verheul and Sjaak Philipsen wrote the paper. All authors critically reviewed and agreed with the paper.

## CONFLICT OF INTEREST STATEMENT

The authors declare no conflict of interest.

## FUNDING

Work in our laboratories was supported by the Landsteiner Foundation for Blood Transfusion Research (1627), Sophia Childrens' Hospital Foundation (WAR20‐21), Erasmus MC Human Disease Models award (108842), TKI Health Holland (EMCLSH20025), ZonMW PSIDER consortium TRACER (10250022110001), and NWO Applied and Engineering Sciences Open Technology Programme (18947).

## Supporting information

Supporting information.

Supporting information.

## Data Availability

The data that support the findings of this study are openly available in the European Nucleotide Archive at https://www.ebi.ac.uk/ena/browser/search, reference number PRJEB67342 and PRJEB31728.

## References

[1] Weatherall D. The inherited disorders of haemoglobin: an increasingly neglected global health burden. Indian J Med Res. 2011;134:493‐497. 22089613 PMC3237249

[2] Houwing ME , de Pagter PJ , van Beers EJ , et al. Sickle cell disease: Clinical presentation and management of a global health challenge. Blood Rev. 2019;37:100580. 10.1016/j.blre.2019.05.004 31128863

[3] Watson J. , Starman A.W. , Bilello F.P. The significance of the paucity of sickle cells in newborn Negro infants. Am J Med Sci. 1948;215(4):419‐423. 10.1097/00000441-194804000-00008 18107723

[4] Fessas P , Stamatoyannopoulos G. Hereditary persistence of fetal hemoglobin in Greece. A study and a comparison. Blood. 1964;24:223‐240. 14214133

[5] Musallam KM , Taher AT , Cappellini MD , Sankaran VG. Clinical experience with fetal hemoglobin induction therapy in patients with β‐thalassemia. Blood. 2013;121(12):2199‐2212. 10.1182/blood-2012-10-408021 23315167

[6] Steinberg MH. Fetal hemoglobin in sickle cell anemia. Blood. 2020;136(21):2392‐2400. 10.1182/blood.2020007645 32808012 PMC7685210

[7] Frangoul H , Altshuler D , Cappellini MD , et al. CRISPR‐Cas9 gene editing for sickle cell disease and β‐thalassemia. N Engl J Med. 2021;384(3):252‐260. 10.1056/NEJMoa2031054 33283989

[8] Frangoul H , Locatelli F , Sharma A , et al. Exagamglogene autotemcel for severe sickle cell disease. N Engl J Med. 2024;390(18):1649‐1662. 10.1056/NEJMoa2309676 38661449

[9] Locatelli F , Lang P , Wall D , et al. Exagamglogene autotemcel for transfusion‐dependent β‐thalassemia. N Engl J Med. 2024;390(18):1663‐1676. 10.1056/NEJMoa2309673 38657265

[10] Piel FB , Rees DC , DeBaun MR , et al. Defining global strategies to improve outcomes in sickle cell disease: a Lancet Haematology Commission. Lancet Haematol. 2023;10(8):e633‐e686. 10.1016/S2352-3026(23)00096-0 37451304 PMC11459696

[11] Rankine‐Mullings AE , Nevitt SJ. Hydroxyurea (hydroxycarbamide) for sickle cell disease. Cochrane Database Syst Rev. 2022;9(9):002202. 10.1002/14651858.CD002202.pub3 PMC9435593 36047926

[12] Badat M , Davies J. Gene therapy in a patient with sickle cell disease. N Engl J Med. 2017;376(21):2093‐2094. 10.1056/NEJMc1704009 28541013

[13] Verheul TCJ , Trinh VT , Vázquez O , Philipsen S. Targeted protein degradation as a promising tool for epigenetic upregulation of fetal hemoglobin. ChemMedChem. 2020;15(24):2436‐2443. 10.1002/cmdc.202000574 33002296 PMC7756256

[14] Breveglieri G , Salvatori F , Finotti A , et al. Development and characterization of cellular biosensors for HTS of erythroid differentiation inducers targeting the transcriptional activity of γ‐globin and β‐globin gene promoters. Anal Bioanal Chem. 2019;411(29):7669‐7680. 10.1007/s00216-019-01959-z 31273412

[15] Vadolas J , Wardan H , Orford M , et al. Development of sensitive fluorescent assays for embryonic and fetal hemoglobin inducers using the human β‐globin locus in erythropoietic cells. Blood. 2002;100(12):4209‐4216. 10.1182/blood-2001-12-0365 12393613

[16] Chan KSK , Xu J , Wardan H , McColl B , Orkin S , Vadolas J. Generation of a genomic reporter assay system for analysis of γ‐ and β‐globin gene regulation. FASEB J. 2012;26(4):1736‐1744. 10.1096/fj.11-199356 22267339 PMC4050337

[17] Haley JD , Smith DE , Schwedes J , et al. Identification and characterization of mechanistically distinct inducers of γ‐globin transcription. Biochem Pharmacol. 2003;66(9):1755‐1768. 10.1016/s0006-2952(03)00542-2 14563486 PMC1351252

[18] Skarpidi E , Vassilopoulos G , Li Q , Stamatoyannopoulos G. Novel in vitro assay for the detection of pharmacologic inducers of fetal hemoglobin. Blood. 2000;96(1):321‐326. 10891468

[19] Kurita R , Suda N , Sudo K , et al. Establishment of immortalized human erythroid progenitor cell lines able to produce enucleated red blood cells. PLoS One. 2013;8(3):e59890. 10.1371/journal.pone.0059890 23533656 PMC3606290

[20] Heshusius S , Heideveld E , Burger P , et al. Large‐scale in vitro production of red blood cells from human peripheral blood mononuclear cells. Blood Adv. 2019;3(21):3337‐3350. 10.1182/bloodadvances.2019000689 31698463 PMC6855111

[21] Benowitz AB , Eberl HC , Erickson‐Miller CL , et al. A hit deconstruction approach for the discovery of fetal hemoglobin inducers. Bioorg Med Chem Lett. 2018;28(23‐24):3676‐3680. 10.1016/j.bmcl.2018.10.032 30554630

[22] Makino T , Haruyama M , Katayama K , et al. Phenotypic‐screening generates active novel fetal globin‐inducers that downregulate Bcl11a in a monkey model. Biochem Pharmacol. 2020;171:113717. 10.1016/j.bcp.2019.113717 31751536

[23] Gallego‐Murillo JS , Yağcı N , Pinho EM , Wahl SA , van den Akker E , von Lindern M. Iron‐loaded deferiprone can support full hemoglobinization of cultured red blood cells. Sci Rep. 2023;13(1):6960. 10.1038/s41598-023-32706-1 37117329 PMC10147612

[24] Heshusius S , Grech L , Gillemans N , et al. Epigenomic analysis of KLF1 haploinsufficiency in primary human erythroblasts. Sci Rep. 2022;12(1):336. 10.1038/s41598-021-04126-6 35013432 PMC8748495

[25] van den Akker E , Satchwell TJ , Pellegrin S , Daniels G , Toye AM. The majority of the in vitro erythroid expansion potential resides in CD34(−) cells, outweighing the contribution of CD34(+) cells and significantly increasing the erythroblast yield from peripheral blood samples. Haematologica. 2010;95(9):1594‐1598. 10.3324/haematol.2009.019828 20378567 PMC2930963

[26] Brinkman EK , Chen T , Amendola M , van Steensel B. Easy quantitative assessment of genome editing by sequence trace decomposition. Nucleic Acids Res. 2014;42(22):e168. 10.1093/nar/gku936 25300484 PMC4267669

[27] Concordet JP , Haeussler M. CRISPOR: intuitive guide selection for CRISPR/Cas9 genome editing experiments and screens. Nucleic Acids Res. 2018;46(W1):W242‐W245. 10.1093/nar/gky354 29762716 PMC6030908

[28] Sanjana NE , Shalem O , Zhang F. Improved vectors and genome‐wide libraries for CRISPR screening. Nat Methods. 2014;11(8):783‐784. 10.1038/nmeth.3047 25075903 PMC4486245

[29] Wessels MW , Cnossen MH , van Dijk TB , et al. Molecular analysis of the erythroid phenotype of a patient with BCL11A haploinsufficiency. Blood Adv. 2021;5(9):2339‐2349. 10.1182/bloodadvances.2020003753 33938942 PMC8114548

[30] Kim D , Langmead B , Salzberg SL. HISAT: a fast spliced aligner with low memory requirements. Nat Methods. 2015;12(4):357‐360. 10.1038/nmeth.3317 25751142 PMC4655817

[31] Anders S , Pyl PT , Huber W. HTSeq—a Python framework to work with high‐throughput sequencing data. Bioinformatics. 2015;31(2):166‐169. 10.1093/bioinformatics/btu638 25260700 PMC4287950

[32] Korporaal A , Gillemans N , Heshusius S , et al. Hemoglobin switching in mice carrying the Klf1(Nan) variant. Haematologica. 2021;106(2):464‐473. 10.3324/haematol.2019.239830 32467144 PMC7849558

[33] Subramanian A , Narayan R , Corsello SM , et al. A next‐generation connectivity map: L1000 platform and the first 1,000,000 profiles. Cell. 2017;171(6):1437‐1452. 10.1016/j.cell.2017.10.049 29195078 PMC5990023

[34] Livak KJ , Schmittgen TD. Analysis of relative gene expression data using real‐time quantitative PCR and the 2^−ΔΔCT^ method. Methods. 2001;25(4):402‐408. 10.1006/meth.2001.1262 11846609

[35] Antoniani C , Meneghini V , Lattanzi A , et al. Induction of fetal hemoglobin synthesis by CRISPR/Cas9‐mediated editing of the human β‐globin locus. Blood. 2018;131(17):1960‐1973. 10.1182/blood-2017-10-811505 29519807

[36] Schwinn MK , Machleidt T , Zimmerman K , et al. CRISPR‐mediated tagging of endogenous proteins with a luminescent peptide. ACS Chem Biol. 2018;13(2):467‐474. 10.1021/acschembio.7b00549 28892606

[37] Vinjamur DS , Bauer DE. Growing and genetically manipulating human umbilical cord blood‐derived erythroid progenitor (HUDEP) cell lines. Methods Mol Biol. 2018;1698:275‐284. 10.1007/978-1-4939-7428-3_17 29076097

[38] Moir‐Meyer G , Cheong PL , Olijnik AA , et al. Robust CRISPR/Cas9 genome editing of the HUDEP‐2 erythroid precursor line using plasmids and single‐stranded oligonucleotide donors. Methods Protoc. 2018;1(3):28. 10.3390/mps1030028 31164570 PMC6481050

[39] Sankaran VG , Menne TF , Xu J , et al. Human fetal hemoglobin expression is regulated by the developmental stage‐specific repressor BCL11A. Science. 2008;322(5909):1839‐1842. 10.1126/science.1165409 19056937

[40] Masuda T , Wang X , Maeda M , et al. Transcription factors LRF and BCL11A independently repress expression of fetal hemoglobin. Science. 2016;351(6270):285‐289. 10.1126/science.aad3312 26816381 PMC4778394

[41] Grevet JD , Lan X , Hamagami N , et al. Domain‐focused CRISPR screen identifies HRI as a fetal hemoglobin regulator in human erythroid cells. Science. 2018;361(6399):285‐290. 10.1126/science.aao0932 30026227 PMC6257981

[42] Liu L , Pertsemlidis A , Ding LH , et al. Original research: a case‐control genome‐wide association study identifies genetic modifiers of fetal hemoglobin in sickle cell disease. Exp Biol Med. 2016;241(7):706‐718. 10.1177/1535370216642047 PMC4950389 27022141

[43] Wongborisuth C , Chumchuen S , Sripichai O , et al. Down‐regulation of the transcriptional repressor ZNF802 (JAZF1) reactivates fetal hemoglobin in β0‐thalassemia/HbE. Sci Rep. 2022;12(1):4952. 10.1038/s41598-022-08920-8 35322124 PMC8943019

[44] Qin K , Lan X , Huang P , et al. Molecular basis of polycomb group protein‐mediated fetal hemoglobin repression. Blood. 2023;141(22):2756‐2770. 10.1182/blood.2022019578 36893455 PMC10273169

[45] Yu L , Myers G , Schneider E , et al. Identification of novel γ‐globin inducers among all potential erythroid druggable targets. Blood Adv. 2022;6(11):3280‐3285. 10.1182/bloodadvances.2021006802 35240686 PMC9198928

[46] Traxler EA , Yao Y , Wang YD , et al. A genome‐editing strategy to treat β‐hemoglobinopathies that recapitulates a mutation associated with a benign genetic condition. Nat Med. 2016;22(9):987‐990. 10.1038/nm.4170 27525524 PMC5706766

[47] Gilman JG , Mishima N , Wen XJ , Stoming TA , Lobel J , Huisman T.H.J. Distal CCAAT box deletion in the A globin gene of two black adolescents with elevated fetal A globin. Nucleic Acids Res. 1988;16(22):10635‐10642. 10.1093/nar/16.22.10635 2462713 PMC338929

[48] Wu Y , Zeng J , Roscoe BP , et al. Highly efficient therapeutic gene editing of human hematopoietic stem cells. Nat Med. 2019;25(5):776‐783. 10.1038/s41591-019-0401-y 30911135 PMC6512986

[49] Dulmovits BM , Appiah‐Kubi AO , Papoin J , et al. Pomalidomide reverses γ‐globin silencing through the transcriptional reprogramming of adult hematopoietic progenitors. Blood. 2016;127(11):1481‐1492. 10.1182/blood-2015-09-667923 26679864 PMC4797024

[50] DeSimone J , Koshy M , Dorn L , et al. Maintenance of elevated fetal hemoglobin levels by decitabine during dose interval treatment of sickle cell anemia. Blood. 2002;99(11):3905‐3908. 10.1182/blood.v99.11.3905 12010787

[51] Meiler SE , Wade M , Kutlar F , et al. Pomalidomide augments fetal hemoglobin production without the myelosuppressive effects of hydroxyurea in transgenic sickle cell mice. Blood. 2011;118(4):1109‐1112. 10.1182/blood-2010-11-319137 21536862 PMC3148160

[52] Moutouh‐de Parseval LA , Verhelle D , Glezer E , et al. Pomalidomide and lenalidomide regulate erythropoiesis and fetal hemoglobin production in human CD34+ cells. J Clin Invest. 2008;118(1):248‐258. 10.1172/JCI32322 18064299 PMC2117764

[53] Krivega I , Byrnes C , de Vasconcellos JF , et al. Inhibition of G9a methyltransferase stimulates fetal hemoglobin production by facilitating LCR/γ‐globin looping. Blood. 2015;126(5):665‐672. 10.1182/blood-2015-02-629972 25979948 PMC4520881

[54] Nualkaew T , Khamphikham P , Pongpaksupasin P , et al. UNC0638 induces high levels of fetal hemoglobin expression in β‐thalassemia/HbE erythroid progenitor cells. Ann Hematol. 2020;99(9):2027‐2036. 10.1007/s00277-020-04136-w 32567028

[55] Renneville A , Van Galen P , Canver MC , et al. EHMT1 and EHMT2 inhibition induces fetal hemoglobin expression. Blood. 2015;126(16):1930‐1939. 10.1182/blood-2015-06-649087 26320100 PMC4608240

[56] Takase S , Hiroyama T , Shirai F , et al. A specific G9a inhibitor unveils BGLT3 lncRNA as a universal mediator of chemically induced fetal globin gene expression. Nat Commun. 2023;14(1):23. 10.1038/s41467-022-35404-0 36635268 PMC9837035

[57] Migliaccio AR , Rotili D , Nebbioso A , Atweh G , Mai A. Histone deacetylase inhibitors and hemoglobin F induction in β‐thalassemia. Int J Biochem Cell Biol. 2008;40(11):2341‐2347. 10.1016/j.biocel.2008.04.024 18617435 PMC2581454

[58] Pourfarzad F , von Lindern M , Azarkeivan A , et al. Hydroxyurea responsiveness in β‐thalassemic patients is determined by the stress response adaptation of erythroid progenitors and their differentiation propensity. Haematologica. 2013;98(5):696‐704. 10.3324/haematol.2012.074492 23100274 PMC3640112

[59] Ley TJ , DeSimone J , Anagnou NP , et al. 5‐Azacytidine selectively increases γ‐globin synthesis in a patient with β+thalassemia. N Engl J Med. 1982;307(24):1469‐1475. 10.1056/NEJM198212093072401 6183586

[60] Molokie R , Lavelle D , Gowhari M , et al. Oral tetrahydrouridine and decitabine for non‐cytotoxic epigenetic gene regulation in sickle cell disease: a randomized phase 1 study. PLoS Med. 2017;14(9):e1002382. 10.1371/journal.pmed.1002382 28880867 PMC5589090

[61] Ali Z , Ismail M , Rehman IU , Rani GF , Ali M , Khan MTM. Long‐term clinical efficacy and safety of thalidomide in patients with transfusion‐dependent β‐thalassemia: results from Thal‐Thalido study. Sci Rep. 2023;13(1):13592. 10.1038/s41598-023-40849-4 37604857 PMC10442319

[62] Ansari SH , Ansari I , Wasim M , et al. Evaluation of the combination therapy of hydroxyurea and thalidomide in β‐thalassemia. Blood Adv. 2022;6(24):6162‐6168. 10.1182/bloodadvances.2022007031 35477175 PMC9772794

[63] Bhattacharjee U , Khadwal A , Shafiq N , et al. A phase 2 randomized controlled trial of single‐agent hydroxyurea versus thalidomide among adult transfusion dependent β thalassemia patients. Indian J Hematol Blood Transf. 2023;39(2):266‐275. 10.1007/s12288-022-01620-3 PMC9809516 36620489

[64] Garg A , Patel K , Shah K , et al. Safety and efficacy of thalidomide and hydroxyurea combination in β‐thalassemia patients. Indian J Hematol Blood Transf. 2023;39(1):85‐89. 10.1007/s12288-022-01536-y PMC9868204 36699430

[65] Li H , Xie W , Gore ER , et al. Development of phenotypic screening assays for γ‐globin induction using primary human bone marrow day 7 erythroid progenitor cells. SLAS Discovery. 2013;18(10):1212‐1222. 10.1177/1087057113499776 24163393

[66] Esrick EB , Lehmann LE , Biffi A , et al. Post‐transcriptional genetic silencing of BCL11A to treat sickle cell disease. N Engl J Med. 2021;384(3):205‐215. 10.1056/NEJMoa2029392 33283990 PMC7962145

[67] Gambari R , Waziri AD , Goonasekera H , Peprah E. Pharmacogenomics of drugs used in β‐thalassemia and sickle‐cell disease: from basic research to clinical applications. Int J Mol Sci. 2024;25(8):4263. 10.3390/ijms25084263 38673849 PMC11050010

[68] Gu Q , Palani CD , Smith A , et al. MicroRNA29B induces fetal hemoglobin via inhibition of the HBG repressor protein MYB in vitro and in humanized sickle cell mice. Front Med. 2022;9:1043686. 10.3389/fmed.2022.1043686 PMC9732025 36507536

