# Supplementary material for: A cellular reporter system to evaluate endogenous fetal hemoglobin induction and screen for therapeutic compounds
Source: Hemasphere. 2024 Aug 6;8(8):e139. doi: 10.1002/hem3.139 (PMC11302795; doi:10.1002/hem3.139)
Supplement: Supplementary file 1 — Supporting information. [file HEM3-8-e139-s001.pdf]

Supplementary Information

Supplementary Materials and Methods

Sanger sequencing

To isolate genomic DNA, approximately 500,000 HUDEP or HEK293T cells were resuspended in lysis buffer (100 mM Tris-HCL pH 8.0, 200 mM NaCl, 5 mM EDTA, 0.2% SDS and 40µg/mL proteinase K). DNA was obtained from the lysate by isopropanol precipitation and quantified with the NanoDrop 2000 spectrophotometer (Thermo Fisher Scientific Inc, Waltham, MA). Polymerase chain reactions (PCR) were performed in the Professional Standard Gradient 96 thermocycler (Biometra, Göttingen, DE, catalogue #070-851). The total volume of the reaction mixture was 25 µL per condition and contained approximately 25 ng gDNA; 0.1 µL Platinum Taq DNA Polymerase with 2.5 µL 10x PCR Rxn Buffer and 0.75 µL 50mM MgCl2 (Kit from Thermo Fisher Scientific, catalogue #10966034); 0.5 µL 20mM dNTPs and 0.5 µL 10mM of both the forward and the reverse primer (**Table S1**). Primers were used at a final concentration of 0.2 µM each. The standard PCR program for the amplicons below 1kb in length, started with 94°C for 2 minutes followed by 25 cycles of 94°C for 30 seconds, 58°C for 30 seconds and 72°C for 45 seconds, followed by a final step of 68°C for 2 minutes. For longer amplicons the PCR program was extended, e.g. the 5 kb program contained 30 cycles of 94°C for 30 seconds, 58°C for 30 seconds and 72°C for 5 minutes. PCR products were run on agarose gels supplemented with ethidium bromide (0.5 µg/mL) at 120V for 1 hour (1.2% agarose for amplicons of approximately 500 bases - 0.7% agarose for 5kb amplicons) and subsequently visualized with UV-light and compared to the Lambda DNA/PstI marker to estimate amplicon length. All DNA purification, from agarose gel or directly after PCR, was performed using the NucleoSpin Gel and PCR Clean-up kit (Macherey-Nagel, Düren, DE, catalogue #740609.250) following manufacturer’s instructions. 5 µL (20-80 ng/µL) purified DNA was premixed with 5 µL (5 pmol/µL) primer and sent to Eurofins Genomics (Ebersberg, DE), for Sanger sequencing. Sequence results were analyzed using the CLC Main Workbench 8 software from Qiagen Bioinformatics (Redwood City, CA). Sanger sequence trace deconvolution to estimate Cas9 efficiency was performed with either the TIDE[1] or the ICE analysis tool[2].

Quantitative PCR

RNA was isolated from HUDEP cells utilizing TRI-Reagent (Sigma-Aldrich, St Louis, MO, catalogue #93289) and chloroform (Sigma-Aldrich, catalogue #25690). RNA concentrations were determined using the NanoDrop 2000 spectrophotometer (Thermo Fisher Scientific) following manufacturer’s instructions. 300-500 ng RNA was then used for cDNA synthesis with Revert aid H minus RT (Thermo Fisher Scientific, catalogue #EP0451). The reactions were performed in white 96-well microplates (Bio-Rad Laboratories, Hercules, CA, catalogue #HSP9635) in CFX96 thermocyclers (Bio-Rad Laboratories, Hercules, CA).

Each 25 µL qPCR reaction mixtures typically contained 0.1 µL Platinum Taq DNA Polymerase with 2.5 µL matching 10× PCR buffer and 0.75 µL MgCl2 (kit from Thermo Fisher Scientific, catalogue #10966034); 0,5 µL dNTP; 0.5 µL 10 µM primer mixture (forward and reverse, see **Table S1**); 0.1 µL SYBR Green; and 0.2 µL cDNA. The qPCR program consisted of 40 three-step cycles (25 seconds 95°C – 25 seconds 60°C – 15 seconds 72°C), preceded by an initial 3:00 minutes at 95°C.

The threshold cycle (Ct) values of the reference gene *PSMD1* were used to normalize Ct values of the hemoglobin subunits. *PSMD1* expression levels remain stable in early differentiation outperforming typical reference gene expression levels that are dependent on cell differentiation status [3-5]. Data was analyzed with Office 2016 Excel (Microsoft, Redmond, WA). The $2^{-\Delta\Delta CT}$ method was used as a relative quantification strategy to calculate relative gene-expression levels from Ct values.[6] To assess total expression levels of the γ-globin subunit, we used a primer pair that amplifies identical regions in *HBG1* and *HBG2*. To distinguish between specific γ-globin induction and induction of β-like subunits we report γ/(γ+β) ratios calculated from $2^{-\Delta CT}$ values.

SDS-Page and Western Blot

Proteins were isolated from 1×10^6 HUDEP2 cells through lysis in RIPA buffer supplemented with 1:100 volume proteinase inhibitor Pefabloc (Sigma-Aldrich, catalogue #11429868001) and 1:100 volume protease inhibitor (Sigma-Aldrich, catalogue #4719956001). Proteins were boiled for 5 minutes in a 2:1 ratio with 2x Laemmli buffer containing β-mercaptoethanol (Sigma-Aldrich, catalogue #S3401). Samples were loaded on a freshly prepared polyacrylamide gel (6.25%) and were run in Tris-Glycine-SDS buffer (Bio-Rad Laboratories, catalogue #1610732). Transfer onto nitrocellulose membranes (GE Healthcare Life Sciences, Freiburg, DE, catalogue #10600002) was conducted in Towbin buffer (25 mM Tris; 192 mM glycine) with 10% or 20% methanol (Sigma-Aldrich, catalogue #32213) at 100 V for 1-2 hours. Membranes were blocked in 1-2% BSA in TBS-Tween for 30 minutes - 1 hour at room temperature (Tween: Sigma-Aldrich, catalogue #P1379). Incubation with primary antibodies was performed for 1 hour in TBS-Tween containing 5% BSA. Afterwards, membranes were washed three times with TBS-Tween and incubated with the appropriate secondary antibodies (all used at 1:4,000 dilution) in 5% Blotting-Grade Blocker (Bio-Rad Laboratories, catalogue #170-6404) in TBS-Tween for 1 hour at room temperature. The monoclonal mouse primary antibody for NPM1 (Abcam, Cambridge, UK, ab10530) and the monoclonal mouse primary antibody for γ-globin (Santa Cruz Biotechnology, Santa Cruz, CA, sc-21756) and β-globin (sc-21757, Santa Cruz Biotechnology). The primary antibodies were used at 1:2,000 dilution. Secondary goat anti-mouse antibodies in green (LI-COR, Bad Homburg, DE, catalogue #926-32213) allowed visualization of the proteins of interest on the membranes when analyzed on an Odyssey CLx Imaging System (LI-COR).

Next generation sequencing

Sample preparation for OMNI-ATACseq

The Assay for Transposase Accessible Chromatin coupled to high-throughput sequencing (ATACseq) was performed as previously described[7] with slight modifications. Approximately 50,000 single cells were then lysed in resuspension buffer containing 0.1% IgePal, 0.1% Tween-20 and 0.01% Digitonin. Immediately after lysis, the buffer was washed out by resuspension buffer with only Tween-20. Nuclei were centrifuged at 500xg for 10 min at 4°C. Nuclei were re-suspended in 25 µl 2x TD buffer (Illumina, San Diego, CA), 2.5 µl TDE1 (transposase, Illumina), 16.5 µl PBS, 0.5 µl Tween-20, 0.5 µl Digitonin, and incubated for 30 min at 37°C. Afterwards, the sample was purified using the MinElute PCR Purification Kit (Qiagen) according to the manufacturer's protocol and eluted with 10 µl elution buffer.

Samples were amplified using the NEBNext High Fidelity PCR master mix (New England Biolabs, Ipswich, MA) and afterwards purified using a MinElute PCR purification kit (Qiagen) according to the manufacturer's instructions. One microliter was loaded on an Agilent Technologies (Santa Clara, CA) 2100 Bioanalyzer using a DNA 1000 assay to determine the library concentration and for quality check.

RNAseq sample preparation

Total RNA was checked for quality on an Agilent Technologies 2100 Bioanalyzer using RNA nano assays. All samples had RIN value greater than 8. RNA-seq libraries were prepared according to the Illumina TruSeq stranded mRNA protocol (www.illumina.com). Briefly, 200 ng of total RNA was purified using poly-T oligo-attached magnetic beads to isolate poly-A containing mRNA. The poly-A tailed mRNA was fragmented and cDNA was synthesized using SuperScript II and random primers in the presence of Actinomycin D. cDNA fragments were end repaired, purified with AMPure XP beads, A-tailed using Klenow exo-enzyme in the presence of dATP. Paired-end adapters with dual index (Illumina) were ligated to the A-tailed cDNA fragments and purified using AMPure XP beads. The resulting adapter-modified cDNA fragments were enriched by PCR using Phusion polymerase as followed: 30 s at 98°C, 15 cycles of (10 s at 98°C, 30 s at 60°C, 30 s at 72°C), 5 min at 72°C. PCR products were purified using AMPure XP beads and eluted in 30 µl of resuspension buffer. One microliter was loaded on an Agilent Technologies 2100 Bioanalyzer using a DNA 1000 assay to determine the library concentration and for quality check.

Bridge amplification and Sequencing by synthesis for RNAseq and ATACseq

Cluster generation was performed according to the Illumina TruSeq SR Rapid Cluster kit v2 Reagents Preparation Guide (www.illumina.com). Briefly, for sequencing libraries were pooled to get a stock of 10 nM. One microliter of the 10 nM stock was denaturated with NaOH, diluted to 10 pM and hybridized onto the flow cell. The hybridized products were sequentially amplified, linearized and end-blocked according to the Illumina Single Read Multiplex Sequencing user guide. After hybridization of the sequencing primer, sequencing-by-synthesis was performed using the HiSeq 2500 using single read 50 bases for RNAseq libraries and paired-end 50 for the OMNI-ATAC library. All libraries were sequenced to a minimal depth of $20x10^6$ reads.

OMNI-ATAC Analysis

Illumina adapter sequences were trimmed off the reads, which were subsequently mapped against the GRCh38 human reference using HiSat2 (version 2.1.0) [8] in paired-end mode. Peaks were called using MACS2 (version 2.1.1).[9]

RNAseq data analysis

Cutadapt (doi: 10.14806/ej.17.1.200) was used to trim Illumina adapter sequences from the reads, which were subsequently mapped against the GRCh38 human reference using HiSat2 (version 2.2.1).[8] Gene expression values were called using htseq-count (version 0.12.4)[10] and Ensembl release 101 gene and transcript annotation. Expression values were calculated as Reads per Kilobase per Million fragments (RKPM).

Generation of the HDR templates

In HUDEP1 cells a dsDNA template was used to for the HDR-mediated knockin of eGFP. The T2A-eGFP sequence was PCR-amplified from a modified p.eGFP_C2 plasmid (Addgene (#2488). Up- and downstream *HBG1*-homology arms -each 800bp in length- were PCR amplified from HUDEP2 genomic DNA. Following manufacturer's instructions the components were assembled in pBluescript (-) KS vector (Addgene #1950) with the Gibson assembly kit (New England Biolabs, cat. #E5510S). Sanger sequencing confirmed the correct assembly. The single-stranded template used to generate the *HBG1-HiBiT* reporter fusion in HUDEP2 cells was ordered from Integrated DNA Technologies Inc (Coralville, IA) as was the case for all other oligonucleotides (**Table S1**).

SNP arrays

Genomic DNA extracted from the HUDEP2 cell line and its *HBG1-HiBiT*-tagged derivatives was used for the Global Screening Array (GSAv3) (Illumina). The global screening array was used according to standard protocols, followed by analysis in GenomeStudio software (Illumina). GenomeStudio final reports were used to analyze and visualize the results in Nexus Discovery (BioDiscovery, El Segundo, CA). A report resolution of 50 kb was used to analyze the data for chromosomal aberrations and differences between the parental and tagged cell lines. BioDiscovery's SNP-FASST2 Segmentation Algorithm is an extension of the FASST2 Segmentation Algorithm, a Hidden Markov Model (HMM)-based approach that -unlike other common HMM methods- does not aim to estimate the copy number state at each probe but uses many states to cover more possibilities, such as mosaic events, and then make calls based on a second level threshold. With the SNP-FASST2 algorithm, B-allele frequency probes are assigned to a range of possible states and a combination of the B-Allele Frequency and Log-R states are used to make the final copy number and allelic event calls. The significance threshold for segmentation was set at $5*10^{-7}$ also requiring a minimum of 25 probes per segment and a maximum contiguous probe spacing of 1000 kb between adjacent probes before breaking a segment.

HiBiT signal detection

For the Nano-Glo HiBiT lytic detection assay (Promega, Madison, WI, catalogue #N3030) 20-50 µL of cell suspension was transferred into a white 96-well plate. As a general rule, black plates (Greiner Bio-One, Kremsmünster, AT, catalogue #655077) were used for fluorescence read-out and white plates (Greiner Bio-One, catalogue #655074) for luminescence read-out. An equal volume (20-50 µL) of lytic detection buffer was supplemented with the inactive LgBiT and its substrate and then added to the cell suspension. After 10 minutes incubation the plate was interrogated with a GloMax plate reader

(Promega). Results were analyzed in Office 2016 Excel (Microsoft) or Graphpad Prism (GraphPad Software, Boston, MA). For the Nano-Glo HiBiT blotting assay (Promega, catalogue #N2410) cell lysates were run on SDS-PAGE gels and blotted as described above followed by probing of the membrane following manufacturer's instructions.

gRNA design

After PCR amplification and sequencing of the 3' end of *HBG1* and *HBG2*, the sequences were compared using the NCBI Basic Local Alignment Search Tool (BLAST). This way gene-specific nucleotides were identified. The CRISPOR tool was utilized to identify Protospacer Adjacent Motifs (PAM) and potential guides in the sequence of *HBG1*.[11] Two guides containing *HBG1*-specific nucleotides with acceptable predicted efficiency were identified and dubbed gRNA #1 and gRNA #2.

gRNA #1 http://crispor.tefor.net/crispor.py?batchId=qIbyx7LYBl9g1Bmx2bnL&pamId=s81-&pam=NGG
gRNA #2 http://crispor.tefor.net/crispor.py?batchId=qIbyx7LYBl9g1Bmx2bnL&pamId=s73-&pam=NGG

Flow Cytometry

For flow cytometry HUDEP cells were centrifuged for 7 minutes at 100×g. Cell pellets were then resuspended in sterile Dulbecco's Phosphate Buffered Saline (PBS) (Lonza, Basel, CH, catalogue #BE17-512F) and counted on the CASY Model TTT cell counter. The cell suspensions were then centrifuged at 550×g for 5 minutes to remove the PBS and resuspended at a 10×10^6 cells/mL in Hank's Balanced Salt Solution (HBSS) (Thermo Fisher Scientific, catalogue #14185045) supplemented with 3% FCS (Sigma-Aldrich, catalogue #F0804). The cells were filtered through 35 µm mesh cell strainer caps of 5 mL polystyrene round bottom tubes (BD biosciences, San Jose, CA, US, catalogue #352235). Stainings were performed with mouse anti-human CD253a (BD biosciences, catalogue #561775) and mouse anti-human CD117 (BD biosciences, catalogue #562435). When sorting, the walls of 5 mL polypropylene round bottom collection tubes (BD biosciences, catalogue #352063) or Falcon 96-well microtiter plates (Thermo Fisher Scientific, catalogue #353075), were blocked with FCS to prevent the sorted cells from sticking to the wall. The FCS was then taken from the 5 mL tubes leaving 100µl at the bottom for cells to be sorted in. In the case of 96-well plates, single cells were sorted into 100 µL proliferation medium. FACS was performed with FACSARIA III (BD biosciences) at the Erasmus MC FACS Sorting Shared Facility. Flow cytometry was performed with the LSR-FORTESSA (BD biosciences). Flow Cytometry data was analyzed with the Flowjo software v.10.6 (BD biosciences).

Nucleofection

For nucleofection 2×10^6 cells per condition were centrifuged (550×g for 5 minutes) after which all the medium was removed and the cell pellets were re-suspended in 100 µL Primary Cell Solution from the Amaxa P3 Primary Cell 4D-Nucleofector kit (Lonza, catalogue #V4XP-3012). The conditioned medium was collected for use after nucleofection. The EW-113 Program 1 was used on the 4D-Nucleofector system (Lonza). After nucleofection the cells were incubated at room temperature in the cuvette for 10 minutes in the ATP-containing nucleofection buffer, after which 400 µL conditioned medium was added.

The 500 µL mixture was then transferred to a 6-well plate with 1.5 mL fresh proliferation medium per well. For each nucleofection experiment, the pmaxGFP (provided with the kit) was taken along as a control. The control cells were checked for GFP-signal under an Olympus IX70 fluorescence microscope (Olympus, Tokyo, JP) and / or through flow cytometry on a BD LSR Fortessa (BD biosciences) 48 hours post-nucleofection to assess nucleofection efficiency. For the plasmid-based genome editing, per condition 1µg pLentiCRISPR v2 (Addgene #52961)[12] encoding both Cas9 and the desired gRNA sequence (**Table S1**) was added to the mixture with 8 µg double-stranded template DNA. For plasmid-free genome editing experiments, 12.5 µL freshly prepared Cas9-sgRNA_#2 ribonucleoprotein (RNP) was added to the nucleofection mixture with 4.8 µL 100 µM single-stranded template DNA. Alt-R CRISPR-Cas9 reagents were purchased from Integrated DNA Technologies and RNPs were prepared following manufacturer's instructions. In short, 3.75 µL 100 µM sgRNA #2 spacer RNA was mixed with 3.75 µL 100 µM tracrRNA and heated to 95ºC and then cooled on the bench top. When at room temperature, 5 µL 62µM Cas9 was added and incubated for 15 minutes before nucleofection to form RNPs.

Clonal expansion after genome editing

To generate clonal cell lines, the cells were single-cell sorted two days post-nucleofection. FACS was performed as described above and single cells were sorted in Falcon 96-well microtiter plates (Thermo Fisher Scientific, catalogue #353075) containing 100 µL proliferation medium per well. Every 4th day 10 µL proliferation medium with a 10x higher doxycycline concentration was added to each well to make up for evaporated medium and keep the doxycycline levels high enough. In a typical experiment 5-10 plates would be sorted with 10-20 wells per plate reaching 30% confluency. At this point the cell suspension would be divided in two equal portions. The first half was subjected to the HiBiT lytic assay, while the other half would be transferred to a fresh 96-well plate. At 50% confluency, the lines that appeared to contain the HiBiT-tag were transferred to a 24 well plate and from then on the medium was refreshed every 2-3 days. At this point cells could be stored in liquid nitrogen, or expanded for further experiments.

HPLC

Hemoglobin analysis was performed on a dedicated ion-exchange HPLC system (VARIANT II Hemoglobin Testing System, Bio-Rad Laboratories).[5]

HTS pilot experiment

Assay plates were prepared using 384-well white opaque culture plates (Revvity Inc, Waltham, MA, catalogue # 6007680). The compounds from a Repurposing Library with 5632 known bioactive drugs were plated into columns 3 to 22 with the help of a Janus MDT 384 automated workstation (Revvity). For this, 1.25 µl of a 400 µM compound stock solution was transferred into the wells. In columns 23 and 24 the positive control (pomalidomide) and in column 1 and 2 the negative control (DMSO) was added. 50µl of the cell suspension was dispensed into each well of the assay plates. The final compound concentration was 10 µM and contained 0.1% DMSO (v/v). The negative controls consisted of cell

suspensions with 0.1% DMSO (v/v). The assay plates were placed for 72h in a cell culture incubator (Cytomat 10C, Thermo Fisher Scientific) under the following conditions: 37°C, 95% humidity, 5% CO_2. The plates were then removed from the incubator and equilibrated for 30 minutes to room temperature. Then 25 µl of NanoGlo HiBit Lytic reagent (Promega) was added to each well (see HiBit signal detection section). After 20 minutes of incubation in darkness, the luminescence signal was read with an Envision Xcite 2104 plate reader (Revvity, Waltham, MA) with an internal ultrasensitive luminescence detector. Speed for the read-out was 0.1 sec/well. Dispensing of the cell suspension and the HiBit reagent was done with a Multidrop Combi Reagent Dispenser (Thermo Fisher Scientific) using a small-tube 8-channel dispensing cassette.

Data normalization and visualization

For each plate the luminescence signal values of all wells with the negative control (0.1% DMSO (v/v)) were used to generate a median value. This value served as reference for normalization of the signal strength of the compound-containing wells on this plate and the fold-change for each well was calculated as (raw luminescence signal of a well)/(median signal of reference wells). Data analysis was performed with the ActivityBase Suite from IDBS (Woking, UK). The TIBCO Spotfire Analytics platform (Revvity) was used for data visualization. Data plots showed the calculated fold-change on the y-axis while the wells were listed on the x-axis.

Data availability

NGS data are available under accession numbers PRJEB67342 and PRJEB31728. SNP array data are available on request (j.philipsen@erasmusmc.nl).

References

1. Brinkman EK, Chen T, Amendola M, van Steensel B. Easy quantitative assessment of genome editing by sequence trace decomposition. Nucleic Acids Res. 2014;42(22):e168.
2. Conant D, Hsiau T, Rossi N, et al. Inference of CRISPR Edits from Sanger Trace Data. CRISPR J. 2022;5(1):123-130.
3. Korporaal A, Gillemans N, Heshusius S, et al. Hemoglobin switching in mice carrying the Klf1(Nan) variant. Haematologica. 2021;106(2):464-473.
4. Subramanian A, Narayan R, Corsello SM, et al. A Next Generation Connectivity Map: L1000 Platform and the First 1,000,000 Profiles. Cell. 2017;171(6):1437-1452 e1417.
5. Wessels MW, Cnossen MH, van Dijk TB, et al. Molecular analysis of the erythroid phenotype of a patient with BCL11A haploinsufficiency. Blood Adv. 2021;5(9):2339-2349.
6. Livak KJ, Schmittgen TD. Analysis of relative gene expression data using real-time quantitative PCR and the 2(-Delta Delta C(T)) Method. Methods. 2001;25(4):402-408.
7. Buenrostro JD, Giresi PG, Zaba LC, Chang HY, Greenleaf WJ. Transposition of native chromatin for fast and sensitive epigenomic profiling of open chromatin, DNA-binding proteins and nucleosome position. Nat Methods. 2013;10(12):1213-1218.
8. Kim D, Langmead B, Salzberg SL. HISAT: a fast spliced aligner with low memory requirements. Nat Methods. 2015;12(4):357-360.
9. Zhang Y, Liu T, Meyer CA, et al. Model-based analysis of ChIP-Seq (MACS). Genome Biol. 2008;9(9):R137.
10. Anders S, Pyl PT, Huber W. HTSeq--a Python framework to work with high-throughput sequencing data. Bioinformatics. 2015;31(2):166-169.
11. Concordet JP, Haeussler M. CRISPOR: intuitive guide selection for CRISPR/Cas9 genome editing experiments and screens. Nucleic Acids Res. 2018;46(W1):W242-W245.
12. Sanjana NE, Shalem O, Zhang F. Improved vectors and genome-wide libraries for CRISPR screening. Nat Methods. 2014;11(8):783-784.

Fig. S1

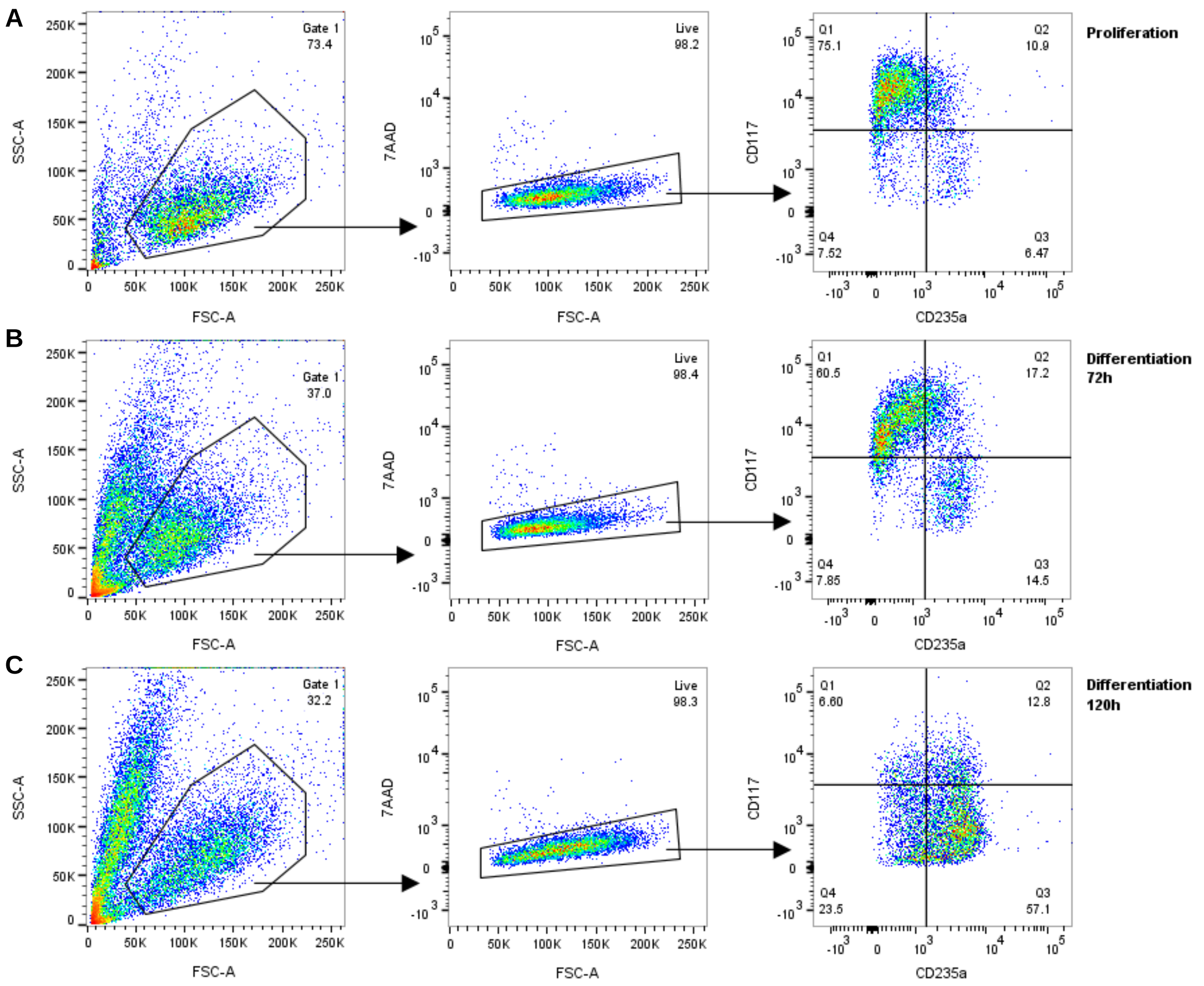

Figure S1. Gating strategy for flow cytometry analysis

Cells were either cultured in **A.** proliferation medium, **B.** differentiation medium for 72h or **C.** differentiation medium for 120h. Cells were stained with 7AAD and CD117 and CD235a antibodies. Left panel: The viable cell population was first gated excluding the majority of cell debris. Middle panel: 7AAD live/dead staining confirmed that >98% of selected cells were alive. Right panel: plots of CD117 *versus* CD235a staining. During differentiation CD117 was lost, while the cells increased CD235a expression.
